# Gut microbiota-derived 3-phenylpropionic acid promotes intestinal epithelial barrier function via AhR signaling

**DOI:** 10.1186/s40168-023-01551-9

**Published:** 2023-05-08

**Authors:** Jun Hu, Jianwei Chen, Xiaojian Xu, Qiliang Hou, Jing Ren, Xianghua Yan

**Affiliations:** 1 State Key Laboratory of Agricultural Microbiology, Hubei Hongshan Laboratory, Frontiers Science Center for Animal Breeding and Sustainable Production, College of Animal Sciences and Technology, Huazhong Agricultural University, Wuhan, Hubei 430070 China; 2 The Cooperative Innovation Center for Sustainable Pig Production, Wuhan, Hubei 430070 China; 3 Hubei Provincial Engineering Laboratory for Pig Precision Feeding and Feed Safety Technology, Wuhan, Hubei 430070 China; 4 BGI Research-Qingdao, BGI, Qingdao, 266555 China

**Keywords:** Intestinal epithelial barrier, Fecal microbiota transplantation, *Bacteroides fragilis*, 3-Phenylpropionic acid, Aryl hydrocarbon receptor

## Abstract

**Background:**

The intestinal epithelial barrier confers protection against the intestinal invasion by pathogens and exposure to food antigens and toxins. Growing studies have linked the gut microbiota to the intestinal epithelial barrier function. The mining of the gut microbes that facilitate the function of intestinal epithelial barrier is urgently needed.

**Results:**

Here, we studied a landscape of the gut microbiome of seven pig breeds using metagenomics and 16S rDNA gene amplicon sequencing. The results indicated an obvious difference in the gut microbiome between Congjiang miniature (CM) pigs (a native Chinese breed) and commercial Duroc × [Landrace × Yorkshire] (DLY) pigs. CM finishing pigs had stronger intestinal epithelial barrier function than the DLY finishing pigs. Fecal microbiota transplantation from CM and DLY finishing pigs to germ-free (GF) mice transferred the intestinal epithelial barrier characteristics. By comparing the gut microbiome of the recipient GF mice, we identified and validated *Bacteroides fragilis* as a microbial species that contributes to the intestinal epithelial barrier. *B. fragilis*-derived 3-phenylpropionic acid metabolite had an important function on the enhancement of intestinal epithelial barrier. Furthermore, 3-phenylpropionic acid facilitated the intestinal epithelial barrier by activating aryl hydrocarbon receptor (AhR) signaling.

**Conclusions:**

These findings suggest that manipulation of *B. fragilis* and 3-phenylpropionic acid is a promising strategy for improving intestinal epithelial barrier.

Video Abstract

**Supplementary Information:**

The online version contains supplementary material available at 10.1186/s40168-023-01551-9.

## Background

The intestinal epithelial barrier is vital for maintaining homeostasis in mammals [1]. The intestinal epithelial barrier confers protection against intestinal invasion by pathogens and exposure to food antigens and toxins [2]. Growing evidence has linked the intestinal epithelial barrier disorders to inflammatory bowel disease (IBD), irritable bowel syndrome (IBS), and infectious diarrhea [1]. The dysfunction of intestinal epithelial barrier increases the selective permeability of the intestinal epithelium, causing increased translocation of pathogens, endotoxins, and inflammatory mediators [3]. The three key indicators, including serum diamine oxidase activities, endotoxin levels, and D-lactic acid levels, were widely used to evaluate the intestinal epithelial barrier function [4]. In pig production, intestinal epithelial barrier may be impaired by several stressors, such as early weaning-induced stress and feed change-induced stress [5]. Antibiotics were widely used in preventing the intestinal epithelial barrier damage-associated diseases. However, in-feed antibiotics were gradually banned because of the increased risk of antibiotic-resistant pathogens [6, 7]. Therefore, mining potential strategy for preventing intestinal epithelial barrier dysfunction is urgently needed.

Increasing evidence has demonstrated that gut microbiota has an important role in host physiology [8]. Manipulation of gut microbiota is considered a promising avenue to facilitate host health [9]. Some probiotics (such as *Bifidobacterium* and *Lactobacillus*) enhance the intestinal epithelial barrier function [10–13]. Probiotics promote the intestinal epithelial barrier function, preferentially through upregulating the intestinal epithelial tight junctions, adherence junctions, and gap junctions, that are responsible for the selective permeability of the gut barrier [14]. Recent evidence has revealed that the intestinal epithelial barrier function may be regulated by gut microbial metabolites such as short-chain fatty acids, indole derivatives, bile acids, conjugated fatty acids, and polyamines [15]. Therefore, manipulating of the gut microbiota using probiotics is a promising avenue to enhance intestinal epithelial barrier function.

Native breeds of livestock are increasingly being recognized as important resources because of their disease resistance and stress tolerance, although most farms run on commercial breeds of livestock [16]. Our previous study demonstrated that native Congjiang miniature (CM) piglets have stronger diarrhea resistance than commercial Landrace × Yorkshire (LY) piglets and that diarrhea resistance is associated with the intestinal microbiota [17]. Here, we systematically deciphered the gut microbiome in seven pig breeds and identified an obvious distinction in the gut microbiome between commercial Duroc × [Landrace × Yorkshire] (DLY) pigs and native CM pigs. Our results demonstrated that CM finishing pigs have stronger intestinal epithelial barrier function than the DLY finishing pigs. Fecal microbiota transplantation (FMT) from CM finishing pigs and DLY finishing pigs to germ-free (GF) mice transferred intestinal epithelial barrier characteristics. We screened and validated the *Bacteroides fragilis* as a microbial species that contributes to the intestinal epithelial barrier function. *B. fragilis*-derived 3-phenylpropionic acid metabolite promoted the intestinal epithelial barrier by activating the aryl hydrocarbon receptor (AhR) signaling. Our results suggest a promising avenue for the prevention of intestinal epithelial barrier dysfunction in pigs.

## Methods

### Pig fecal samples collection and microbial genomic DNA extraction

A total of 112 pigs (including 56 weaned piglets and 56 finishing pigs from 7 breeds) were used in this study to dissect the landscape of pig gut microbiome. All weaned piglets (including eight commercial DLY, eight native Tibetan miniature (TM), eight native Laiwu (LW), eight native Shaziling (SZL), eight native CM, eight native Huanjiang miniature (HM), and eight native Ningxiang (NX) piglets) and finishing pigs (including eight DLY, eight TM, eight LW, eight SZL, eight CM, eight HM, and eight NX pigs) ate food and drank water freely. A total 112 fresh feces samples from these 112 pigs were individually collected and immediately frozen in liquid nitrogen. Fecal microbial genomic DNA extraction was conducted as previously described [18].

### Metagenomics sequencing and data analysis

The DNA libraries for the metagenomics of 112 fecal samples from pigs were constructed using kits (Illumina) and were then sequenced on the Hiseq Xten platform (Illumina) using the strategy of PE150. The raw data were filtered to remove the low-quality reads, duplication reads, and adapter contamination reads using the SOAPnuke software. The host genomic sequencing reads were trimmed by Bowtie2 software. The obtained high-quality clean reads were assembled de novo using the IDBA-UD software to generate contigs individually. Open reading frames (ORFs) in contigs were predicted using MetaGeneMark software. The predicted genes from each sample were merged and clustered using CD-Hit software based on the criteria of identity of > 95% and coverage of > 90% to remove redundant genes. The non-redundant (NR) gene abundance profile was constructed using the Salmon software. The gene functions of NR gene set were further annotated based on KEGG database by Diamond software. We used the MEGAN software to determine the species taxonomic classification of each gene using the NR database BLAST results. Species abundance and functional abundance profiles were generated by summing the abundance of the genes. Principal component analysis (PCA) for KEGG orthologous groups (KOs) was conducted using package “ade4” of R software. Shannon index of KOs were analyzed using package “vegan” of R software.

The DNA libraries for the metagenomics of 17 fecal samples from the recipient GF mice were constructed using the kits (BGI) and were then sequenced on the BGISEQ-2000 platform using the strategy of PE150. Raw data were filtered to remove low-quality reads, duplication reads, and adapter contamination reads using the SOAPnuke software, and the host genomic sequencing reads were trimmed using SOAP2. The obtained high-quality clean reads were assembled de novo by MEGAHIT software to individually generate contigs. Open reading frames (ORFs) in contigs were predicted using the MetaGeneMark software. The predicted genes from each sample were merged and clustered using the CD-Hit software to remove redundant genes. We used the MEGAN software to determine the taxonomic classification of each gene using the NR database BLAST results. Species abundance and functional abundance profiles were generated by summing the abundance of the genes. The gene functions of non-redundant gene sets were further annotated based on public databases (including NR and KEGG) using the Diamond software. The Venn diagram for NR genes and histograms for taxonomic compositions were visualized using the R software. Principal coordinates analysis (PCoA) of gut microbial KOs was performed by package “vegan” of R software. The cladogram for microbial taxonomic composition was analyzed using the LEfSe software. Heatmaps for taxonomic composition were generated using the R software. The Circos for KOs was analyzed using the Circos software. Beta diversity was analyzed using package “vegan” of R software, and the results were visualized using scatterplots from the PCoA and the heatmap. The comparison analysis of microbial taxonomic compositions at several levels were conducted using the STAMP software.

### Bacterial 16S rDNA gene amplicon sequencing and data analysis

Bacterial 16S rDNA gene amplicon sequencing of 112 fecal samples from pigs was performed using the following procedure. Briefly, the bacterial V4 region was amplified using the following primers: F, 5’– NNNNNNNNGTGTGCCAGCMGCCGCGGTAA–3’ and R, 5’–GGACTACHVGGGTWTCTAAT–3’. High-throughput sequencing was conducted on the Illumina HiSeq2500 platform using the PE250 strategy. Clean data were obtained from sequencing raw data using a previously described filter method [18]. The paired-end clean reads with overlaps were merged into tags using the FLASH software. These tags were clustered into OTUs using USEARCH with 97% similarity. Based on the ribosomal database project (RDP) database, these OTUs were taxonomically classified using the RDP Classifier software. The observed species index was calculated using the Mothur software and rarefaction curves were drawn using R software. Species accumulation curve was drawn by R software. The Shannon index was calculated using the Mothur software. Beta diversity based on bray curtis distance was analyzed by the QIIME software and shown using a scatterplot from PCoA. The cladogram was constructed based on the LEfSe software.

The bacterial 16S rDNA gene amplicon sequencing of 17 fecal samples from the recipient GF mice and 16 fecal samples from donor pigs was conducted as follows. Briefly, the bacterial V3-V4 region was amplified using the following primers: F, 5’–NNNNNNNNACTCCTACGGGAGGCAGCAG–3’ and R, 5’–GGACTACHVGGGTWTCTAAT–3’. High-throughput sequencing was conducted on the Illumina Hiseq2500 platform using the PE300 strategy. After filtering, clean data were obtained from raw data. We used the FLASH to merge the clean reads into tags. The bacterial tags were then clustered into amplicon sequence variants (ASVs) with 100% sequence similarity using DADA2. Based on the RDP database, the bacterial ASVs were taxonomically classified by the RDP classifier. Alpha diversity (including the Chao and Shannon indices) was calculated using Mothur, and rarefaction curves were generated by the R software. The beta diversity was analyzed using QIIME and was shown through the scatter plot of the PCoA, Unifrac cluster tree, and heatmap, respectively. The Venn diagram based on the ASVs was drawn using R software. A cladogram was constructed based on LEfSe. A heatmap of the Spearman correlation was generated using the R software. Cytoscape software was performed to construct a correlation network diagram. Histograms and heatmaps for taxonomic composition were generated using the R software. The enterotype analysis of fungal communities was conducted using the R software. The GraPhlAn diagram analysis was conducted using the GraPhlAn software. The KEGG pathway and COG function in bacterial community were predicted using the PICRUSt2 software.

### Fecal microbiota transplantation experiments

We transferred the fecal microbiota of pigs to GF mice. Fecal suspension was prepared as previously described [17]. Briefly, fresh feces were collected from health CM and DLY finishing pigs, respectively. A sterile PBS solution was added to the feces and the fecal slurry was mixed in an anaerobic incubator. Subsequently, the fecal slurry was filtered using steel tea strainers and then filtered again using a 0.224-mm stainless cell strainer. Methylene blue staining was used to stain live microbes, which were counted using a light microscope. Finally, the fecal suspension was supplemented with 10% glycerol (final concentration) and frozen in a refrigerator at −80 °C.

A total of 17 GF Kunming (KM) mice of similar weights were randomly divided into two groups. All mice ate the same food and drank same water freely. GF mice in each group were housed in separate sterile isolators. GF mice in group 1 (DLY-R, *n* = 7) were orally administrated a fecal suspension (150 µL, 10^8^ CFU/mL) from DLY finishing pigs every 3 days from 3 to 8 weeks of age. The GF mice in group 2 (CM-R, *n* = 10) were orally administrated a fecal suspension (150 µL, 10^8^ CFU/mL) from CM finishing pigs every 3 days from 3 to 8 weeks of age. Seventeen fresh feces samples from these mice were individually collected and immediately frozen in liquid nitrogen. Fecal microbial genomic DNA extraction was performed as previously described by us [18].

### Measurements of serum diamine oxidase activities, endotoxin levels, and D-lactic acid levels

The serum diamine oxidase activity was measured using an assay kit (Nanjing Jiancheng Bioengineering Institute (NJBI), A088). The serum endotoxin and D-lactic acid levels were measured using ELISA kits (MEIMIAN, MM-0369, and MM-43853).

### Measurements of organ and blood routine indices and serum immunoglobulins levels

After slaughter, the organs (heart, liver, spleen, kidney, and thymus) were weighted immediately. The epididymal fat was weighted and the gut length was measured. The organ weights were normalized to the body weights of the mice. The epididymal fat weights and gut lengths were also normalized to the body weights. Routine blood indices were examined using an automatic hematology analyzer (BC-2800vet, Mindray). The serum immunoglobulins (IgA, IgG, and IgM) and interferon-γ (IFN-γ) levels were measured using the ELISA kits (NJBI, H108-1, H106, H109, and H025), respectively.

### Analyses of intestinal histological morphology, intestinal sIgA levels, and intestinal goblet cell numbers

Hematoxylin and eosin (H&E) staining was used to analyze the intestinal histological morphology. The intestinal sIgA levels were measured using an ELISA kit (NJBI, H108-2). Protein concentration of whole cell lysates (WCLs) was measured by a BCA protein assay kit (Beyotime, P0012). The sIgA levels were normalized to the total protein content of WCLs. Intestinal periodic acid-Schiff (PAS) staining was used to detect goblet cells. Intestinal goblet cell numbers were normalized to the villus-crypt units.

### Measurements of intestinal IL-22 levels

After slaughter, the intestinal tissues were collected from mice. The intestinal IL-22 levels were examined using an ELISA kit (MEIMIAN, MM-0892). The protein concentration of the WCLs was measured by a BCA protein assay kit (Beyotime, P0012). The IL-22 levels were normalized to the total protein content of WCLs.

### Oral gavage of bacterial strains in mice

These bacterial species (including *Bacteroides ovatus* (BNCC, 260545), *Bacteroides fragilis* (BNCC, 336948), and *Akkermansia muciniphila* (BNCC, 341917)) were provided by the BeNa Culture Collection in China. The *B. ovatus* and *B. fragilis* were cultured in a brain heart infusion (BHI) medium supplemented with 5 g/L yeast extract, 5 mg/L hemin, 1 mg/L vitamin K1, and 0.3 g/L L-cysteine hydrochloride under anaerobic conditions at 37 °C. The *A. muciniphila* was cultured in a thiolglycollate medium under anaerobic conditions at 37 °C. A total of 50 specific pathogen free (SPF) KM mice with similar weights were randomly divided into five groups (*n* = 10). The mice in the five groups were orally administrated with sterile PBS (150 µL), a *B. fragilis* suspension (150 µL, 10^8^ CFU/mL), a *B. ovatus* suspension (150 µL, 10^8^ CFU/mL), an *A. muciniphila* suspension (150 µL, 10^8^ CFU/mL), or a suspension containing three bacterial species (150 µL, 10^8^ CFU/mL, containing equal amounts of the three bacterial species), respectively. This experiment was conducted every other day from 3 to 8 weeks of age. All the mice ate food and drank water freely.

### Identification of serum and fecal metabolites using metabolomics

Global metabolite profiling on serum and feces was conducted using ultra-performance liquid chromatography (UPLC) combined with mass spectrometry. Briefly, sample extracts were mixed equally and analyzed by UPLC and quadrupole-time of flight (QTOF)-tandem mass spectrometry (MS/MS) to identify metabolites based on public databases (including Metline, KEGG, HMDB, Massbank, and Nist-MSMS). By integrating the identified metabolite information into the metaware database (MWDB) established previously by Metware, we constructed a new integrated database. Subsequently, the sample extracts were analyzed by UPLC and electrospray ionization (ESI)-triple quadrupole-linear ion trap mass spectrometer (QTRAP)-MS/MS to identify the metabolites based on the new integrated metabolite database. The differential metabolites between the two groups were identified using the criteria of variable importance in projection (VIP) ≥ 1, absolute Log_2_ (fold change) ≥ 1, and *p*-value < 0.05. The scores OPLS-DA plot was generated using package “MetaboAnalystR” of R software to show the difference among samples. The principal component analysis was drawn by R software to show the difference among samples. A volcano plot was drawn using the R software to show the differential metabolites between the two groups. Identified metabolites were further annotated based on the KEGG database. KEGG enrichment analysis was conducted and shown in bubble diagram.

### Oral administration of metabolites and drugs in mice

A total of 95 SPF KM mice with similar weight were used to evaluate the roles of candidate metabolites in intestinal epithelial barrier function. These mice were randomly divided into 19 groups (*n* = 5). The mice in group 1 (Ctrl) were orally administrated with a vehicle (150 µL) every other day. The mice in groups 2 ~ 19 were orally administrated with 18 candidate metabolites (including desoxycortone (YuanyeBio-Technology, B65163), caffeic acid (Macklin, C804975), salicylaldehyde (Macklin, S817504), trimethylamine N-oxide (Macklin, T833724), 1-aminopropan-2-ol (Macklin, D828281), 3-hydroxy-tetradecanoic acid (Macklin, H862427), malonic acid (Macklin, M813041), Nα-acetyl-L-glutamine (Macklin, N801345), L-fucose (Macklin, L809666), 2-hydroxybutanoic acid (Macklin, D862813), 3-phenylpropionic acid (Sangon Biotech, A602088), cinnamic acid (Sangon Biotech, A501967), L-rhamnose (YuanyeBio-Technology, S64831), 3-indolepropionic acid (Sigma, 220027), 2-[(3-oxo-3-phenylpropyl)amino]acetic acid (Aladdin, P171014), 1,5-anhydro-D-glucitol (J&K Scientific, 309742), (R)-(-)-2-phenylpropionic acid (Macklin, R815532), and (R)-3-hydroxybutanoic acid (Macklin, R896558)) every other day (60 mg/kg), respectively. The mouse experiments were conducted from 3 to 8 weeks of age. All mice were provided with the same food and drink water ad libitum.

A total of 32 SPF KM mice with similar weights were used to investigate the role of the AhR signaling in 3-phenylpropionic acid-mediated intestinal epithelial barrier function. These mice were randomly divided into 4 groups (*n* = 8). The mice in group 1 (Ctrl) were orally administrated with a vehicle every other day. The mice in group 2 (3-PPA) were orally administrated with 3-phenylpropionic acid (60 mg/kg; Sangon Biotech, A602088) every other day. The mice in group 3 (SR1) were orally administrated with StemRegenin 1 (20 mg/kg; Selleck, S2858) every other day. The mice in group 4 (3-PPA + SR1) were orally administrated with 3-phenylpropionic acid (60 mg/kg; Sangon Biotech, A602088) and StemRegenin 1 (20 mg/kg) every other day. The mouse experiments were conducted from 3 to 8 weeks of age. All mice were provided with the same food and drink water ad libitum.

### In vivo intestinal permeability assay using fluorescein isothiocyanate–dextran

A total of 16 SPF mice with similar weights were randomly divided into two groups (*n* = 8). The mice in the two groups were orally administrated with sterile PBS (150 µL) or a *B. fragilis* suspension (150 µL, 10^8^ CFU/mL), respectively. This experiment was conducted every other day from 3 to 7 weeks of age. At the end of this experiment, the mice were oral administrated with fluorescein isothiocyanate–dextran 4 KDa (FD4) (200 mg/kg body weight; Sigma Aldrich, FD4). Four hours later, the blood was collected from the mice and then plasma was obtained. The plasma FD4 fluorescence intensity (excitation 485 nm/emission 525 nm) was measured by a fluorescence microplate reader. The plasma FD4 levels was calculated according to the fluorescence intensity and standard curve.

A total of 16 SPF mice were randomly divided into two groups (*n* = 8). The mice in group 1 (Ctrl) were orally administrated with a vehicle or 3-phenylpropionic acid (60 mg/kg; Sangon Biotech, A602088), respectively. This experiment was conducted every other day from 3 to 7 weeks of age. The in vivo intestinal permeability assay using FD4 was conducted as described above.

A total of 32 SPF mice were randomly divided into four groups (*n* = 8). The mice in group 1 (Ctrl) were orally administrated with a vehicle every other day. The mice in group 2 (3-PPA) were orally administrated with 3-phenylpropionic acid (60 mg/kg; Sangon Biotech, A602088) every other day. The mice in group 3 (SR1) were orally administrated with StemRegenin 1 (20 mg/kg; Selleck, S2858) every other day. The mice in group 4 (3-PPA + SR1) were orally administrated with 3-phenylpropionic acid (60 mg/kg; Sangon Biotech, A602088) and StemRegenin 1 (20 mg/kg) every other day. The mouse experiments were conducted from 3 to 7 weeks of age. The in vivo intestinal permeability assay using FD4 was conducted as described above. All mice were provided with the same food and drink water ad libitum.

### In vivo intestinal permeability assay using lactulose/mannitol test

A total of 16 SPF mice were randomly divided into two groups (*n* = 8). The mice in the two groups were orally administrated with sterile PBS (150 µL) or a *B. fragilis* suspension (150 µL, 10^8^ CFU/mL), respectively. This experiment was conducted every other day from 3 to 7 weeks of age. At the end of this experiment, the mice were orally administrated with 250 μL solution containing lactulose (250 mg/ml; J&K Scientific, 598584) and D-mannitol (50 mg/ml; J&K Scientific, 351126). The gavage doses of lactulose and D-mannitol in our study were chosen as previously described [19]. The gavage doses of lactulose and D-mannitol may affect the ratio of lactulose recovery to mannitol recovery in mice according to previous studies [19–23]. The urine samples were collected for next 24 h from the mice. The lactulose and mannitol contents in urine samples were measured using the lactulose assay kit (Megazyme, K-LACTUL) and D-mannitol assay kit (Megazyme, K-MANOL), respectively. The percentage of urinary recovery of lactulose and mannitol was calculated, respectively. Lactulose/mannitol ratio was calculated as the ratio of the two percentages.

A total of 16 SPF mice were randomly divided into two groups (*n* = 8). The mice in group 1 (Ctrl) were orally administrated with a vehicle or 3-phenylpropionic acid (60 mg/kg; Sangon Biotech, A602088), respectively. This experiment was conducted every other day from 3 to 7 weeks of age. The in vivo intestinal permeability assay using lactulose/mannitol test was conducted as described above.

A total of 32 SPF mice were randomly divided into four groups (*n* = 8). The mice in group 1 (Ctrl) were orally administrated with a vehicle every other day. The mice in group 2 (3-PPA) were orally administrated with 3-phenylpropionic acid (60 mg/kg; Sangon Biotech, A602088) every other day. The mice in group 3 (SR1) were orally administrated with StemRegenin 1 (20 mg/kg; Selleck, S2858) every other day. The mice in group 4 (3-PPA + SR1) were orally administrated with 3-phenylpropionic acid (60 mg/kg; Sangon Biotech, A602088) and StemRegenin 1 (20 mg/kg) every other day. The experiment was conducted from 3 to 7 weeks of age. The in vivo intestinal permeability assay using lactulose/mannitol test was conducted as described above. All mice were provided with the same food and drink water ad libitum.

### Immunohistochemistry analysis

Immunohistochemistry was conducted to analyze the jejunal E-Cadherin, ZO-1, Connexin 43, and RegIIIγ expression distribution in mice. These antibodies, including the anti-E-cadherin (Servicebio, GB12083), anti-ZO-1 (Servicebio, GB111402), anti-Connexin 43 (Servicebio, GB12234), and RegIIIγ (Abcam, ab198216) were used in the immunohistochemical analysis. The immunohistochemistry assay was conducted as previously described [24]. The experiments for negative controls were performed by omitting the primary antibody as previously described [25–27]. The mean optical density was analyzed using ImageJ software.

### Western blot

We used the jejunal mucosa to prepare the protein samples for western blot assay, as previously described [28–30]. The jejunal segments samples were collected from the approximately middle positions in the jejunal tracts, as previously described [28, 31]. The mid-jejunal segments were rinsed with PBS solution, and then the jejunal mucosa samples were gently scraped off. WCLs of the jejunal mucosa were prepared in RIPA lysis buffer (Sangon Biotech, C500005). The nuclear and cytoplasmic extraction of the intestinal epithelium was conducted using a kit (Thermo Fisher Scientific, 78833). This western blot assay was conducted as previously described [31]. Primary antibodies included the anti-E-cadherin (Cell Signaling Technology, 14472S), anti-ZO-1 (ABclonal, A11417), anti-Connexin 43 (Proteintech, 26980-1-AP), anti-Lamin B1 (Proteintech, 12987-1-AP), anti-CYP1A1 (Proteintech, 13241-1-AP), anti-AhR (Proteintech, 67785-1-Ig), anti-β-actin (Sigma, A5441), and anti-β-tubulin (Proteintech, 66240-1-Ig). Secondary antibodies included the HRP-conjugated secondary antibodies (Cell Signaling Technology, 7076S and 7074S).

### Statistical analysis

The software (including GraphPad Prism, R, and STAMP) was used for statistical analysis. Detailed descriptions of the statistical methods are provided in the figure legends. Statistical significance was set at *p* < 0.05.

## Results

### A landscape of pig gut microbiome revealed by metagenomics and 16S rDNA gene amplicon sequencing

**Fig. 1:**
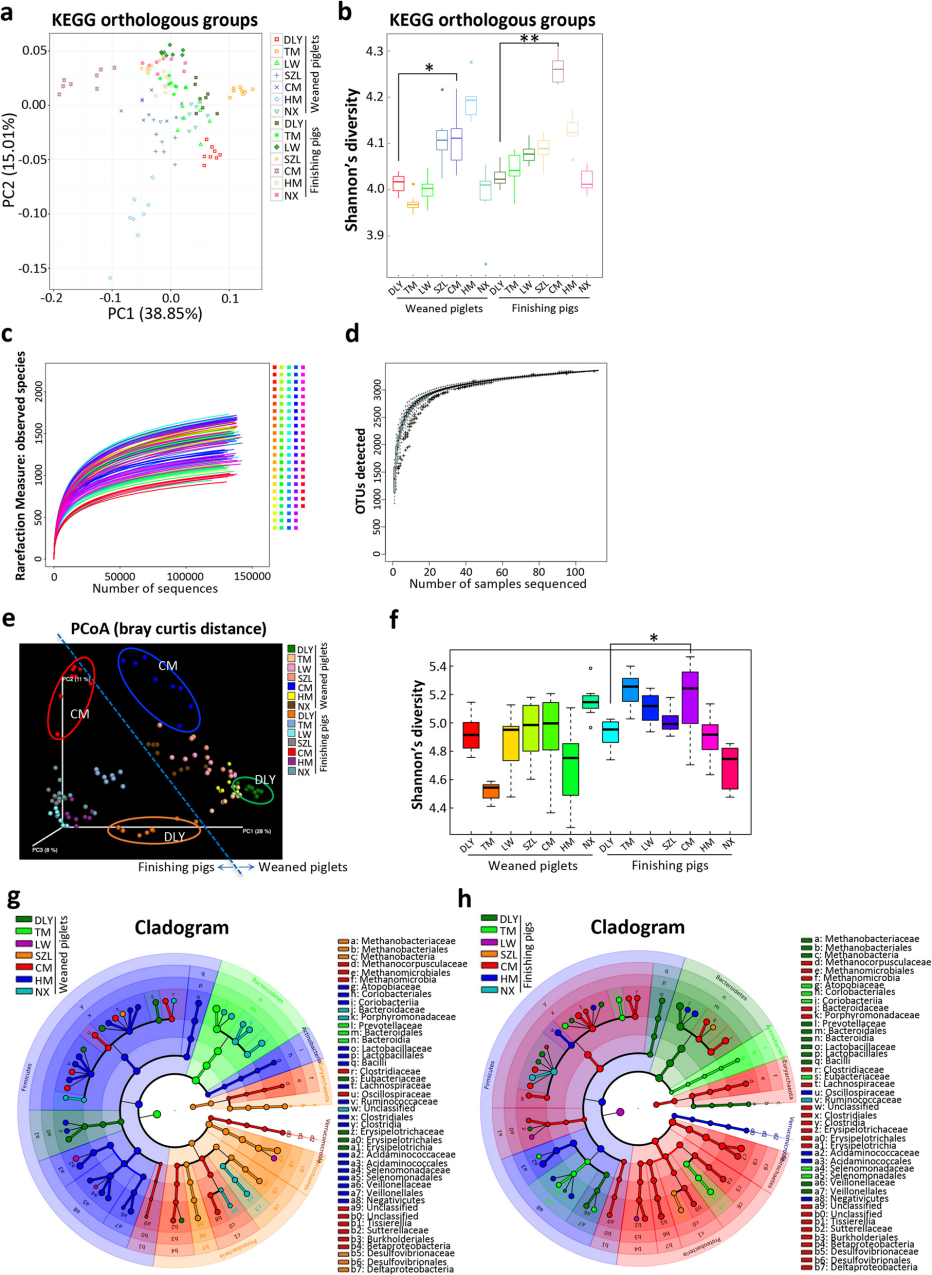
A landscape of pig gut microbiome in China. **a** PCA of gut microbial KEGG orthologous groups (KOs) in pigs revealed by metagenomics. **b** Diversity analysis of KOs based on Shannon index revealed by metagenomics. **c** Rarefaction curves of the observed species index revealed by 16S rDNA gene amplicon sequencing. **d** Species accumulation curve analysis of OTUs revealed by 16S rDNA gene amplicon sequencing. **e** PCoA of gut bacterial community based on the Bray–Curtis distance. **f** Analysis of gut bacterial alpha diversity based on the Shannon index. **g** Cladogram of gut bacterial compositions in weaned piglets using LEfSe. **h** Cladogram of gut bacterial compositions in finishing pigs using LEfSe. The data are presented as the mean ± SEM (*n* = 8) and were evaluated by Kruskal–Wallis test. **p < 0.01, *p < 0.05

To mine the potential probiotics that contribute to intestinal epithelial barrier function, we analyzed the gut microbiome of pigs from seven representative breeds (including commercial DLY pigs, native TM pigs, native LW pigs, native SZL pigs, native CM pigs, native HM pigs, and native NX pigs) in China. Principal component analysis (PCA) of the KEGG orthologous groups (KOs) based on metagenomics showed the differences in gut microbial functional profiles among pig breeds, especially between commercial DLY pigs and native CM pigs (Fig. 1a). The diversity of KOs based Shannon index showed that native CM pigs have significantly higher diversity than commercial DLY pigs (Fig. 1b).

We further used the 16S rDNA gene amplicon sequencing to investigate the gut bacterial community in pigs. Our data showed that the sequencing depth was sufficient to detect the almost all bacterial species, as evidenced by the rarefaction curves (Fig. 1c) and the species accumulation curves (Fig. 1d). Principal coordinates analysis (PCoA) of beta diversity further suggested obvious differences in bacterial communities among pig breeds, especially between commercial DLY pigs and native CM pigs (Fig. 1e). Alpha diversity based on Shannon index also showed differences in gut bacterial communities among the pig breeds (Fig. 1f). The results showed that native CM finishing pigs have significantly higher alpha diversity than commercial finishing DLY pigs (Fig. 1f). Further LEfSe analysis of weaned piglets suggested the differences in bacterial taxonomic compositions among pig breeds (Fig. 1g). LEfSe analysis of finishing pigs indicated that the gut bacterial communities in native CM finishing pigs were mainly enriched with these families, such as Bacteroidaceae, Porphyromonadaceae, and Desulfovibrionaceae (Fig. 1h). Overall, these results showed the differences in the gut microbial functional profiles and taxonomic compositions among the pig breeds, especially between commercial DLY pigs and native CM pigs.

### Fecal microbiota transplantation from pigs to germ-free mice transfers the intestinal epithelial barrier characteristics

**Fig. 2:**
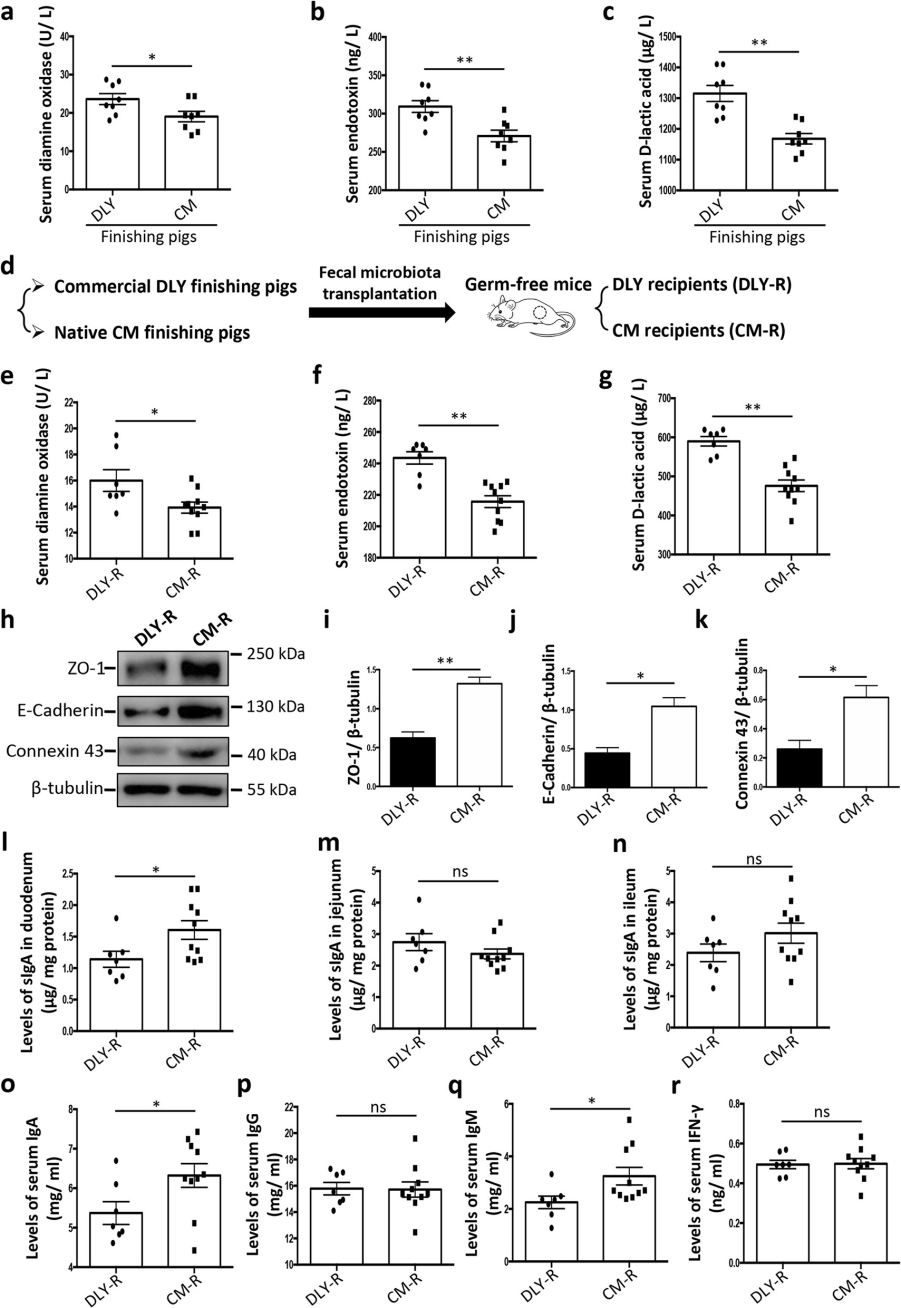
Effects of fecal microbiota transplantation from two pig breeds to germ-free mice on intestinal epithelial barrier function. **a**–**c** The serum diamine oxidase activities (**a**), serum endotoxin levels (**b**), and serum D-lactic acid levels (**c**) in pigs (DLY, Duroc × [Landrace × Yorkshire]; CM, Congjiang miniature). **d** The experimental design of FMT from pigs to GF mice. **e**–**g** Serum diamine oxidase activities (**e**), serum endotoxin levels (**f**), and serum D-lactic acid levels (**g**) in the recipient GF mice (CM-R, the recipient GF mice that received the fecal microbiota from Congjiang miniature pigs; DLY-R, the recipient GF mice that received the fecal microbiota from Duroc × [Landrace × Yorkshire] pigs). **h** Representative western blotting of ZO-1, E-cadherin, Connexin 43, and β-tubulin in the jejunal epithelium of the recipient GF mice. **i**–**k** Quantitation of ZO-1 (**i**), E-cadherin (**j**), and Connexin 43 (**k**) levels normalized to β-tubulin levels. **l**–**n** Levels of sIgA in the duodenum (**l**), jejunum (**m**), and ileum (**n**), respectively. **o**–**r** Levels of serum IgA (**o**), IgG (**p**), IgM (**q**), and IFN-γ (**r**), respectively. The data are presented as the mean ± SEM and evaluated using Student’s *t*-test; *n* = 8 (**a**–**c**); *n* = 10 (CM-R group in **e**–**g** and **l**–**r**) and *n* = 7 (DLY-R group in **e**–**g** and **l**–**r**); *n* = 3 (**i**–**k**). **p < 0.01, *p < 0.05; ns not significant

Given an obvious difference in the gut microbiome between the commercial DLY pigs and native CM pigs, our data suggested a potential difference in the contribution of gut microbiota to host physiology between commercial DLY pigs and native CM pigs. Considering that intestinal epithelial barrier function is critical for host homeostasis maintenance and may be mediated by gut microbiota, we compared the intestinal epithelial barrier function between commercial finishing DLY pigs and native CM finishing pigs. The results showed that the serum diamine oxidase activities, endotoxin levels, and D-lactic acid levels (three key indicators of intestinal epithelial barrier function) were lower in CM finishing pigs than those in DLY finishing pigs (Fig. 2a–c), indicating that CM finishing pigs have a stronger intestinal epithelial barrier function than DLY finishing pigs. Thus, these findings suggest a potential link between gut microbiome and intestinal epithelial barrier function in pigs.

Next, we investigated whether gut microbiota has a regulatory role in intestinal epithelial barrier. To confirm the dominant role of gut microbiota and rule out the genetic influence, we transferred fecal microbiota from native CM finishing pigs and commercial DLY finishing pigs to GF mice, respectively (Fig. 2d). The results demonstrated that the serum diamine oxidase activities, endotoxin levels, and D-lactic acid levels in the recipient GF mice that received the fecal microbiota of CM pigs (CM-R group) were lower than those in the recipient GF mice that received the fecal microbiota of DLY pigs (DLY-R group) (Fig. 2e–g). The levels of intestinal epithelial ZO-1 (a tight junction protein), E-cadherin (an adhesion junction protein), and Connexin 43 (a gap junction protein), that are responsible for selective gut barrier permeability and vital for intestinal epithelial barrier, were higher in the CM-R group than those in DLY-R group (Fig. 2h–k). These findings revealed that the CM-R group had a stronger intestinal epithelial barrier function than the DLY-R group.

Furthermore, our results showed that the CM-R group had higher levels of secretory IgA in the duodenum than the DLY-R group (Fig. 2l–n). The levels of serum IgA and IgM in CM-R group were higher than those in DLY-R group (Fig. 2o–r). The number of peripheral immune cells (including white blood cells, lymphocytes, monocytes, and neutrophils) was not significantly different between the CM-R and DLY-R groups (Additional file 1: Fig. S1a-d). Additionally, there was no significant difference in the number of peripheral red blood cells and blood platelets and the levels of peripheral hemoglobin between the CM-R and the DLY-R groups (Additional file 1: Fig. S1e-g). The kidney index was higher in the CM-R group than that in the DLY-R group, whereas other organ indices (including the heart, liver, spleen, and thymus) were not significantly different between the CM-R and DLY-R groups (Additional file 1: Fig. S1h-l). Neither the epididymal fat nor the gut length indices were significantly different between the CM-R and DLY-R groups (Additional file 1: Fig. S1m, n). The intestinal morphology analysis showed that neither the villus height nor the crypt depth was significantly different between the CM-R and DLY-R groups (Additional file 2: Fig. S2). Moreover, the number of intestinal goblet cells was not significantly different between the CM-R and DLY-R groups (Additional file 3: Fig. S3). Overall, these results showed that FMT from the commercial DLY pigs and native CM pigs to GF mice transferred the intestinal epithelial barrier characteristics, suggesting a critical role of gut microbiota in intestinal epithelial barrier function.

### Comparison analysis of gut microbiome in the recipient germ-free mice after fecal microbiota transplantation revealed by metagenomics

**Fig. 3:**
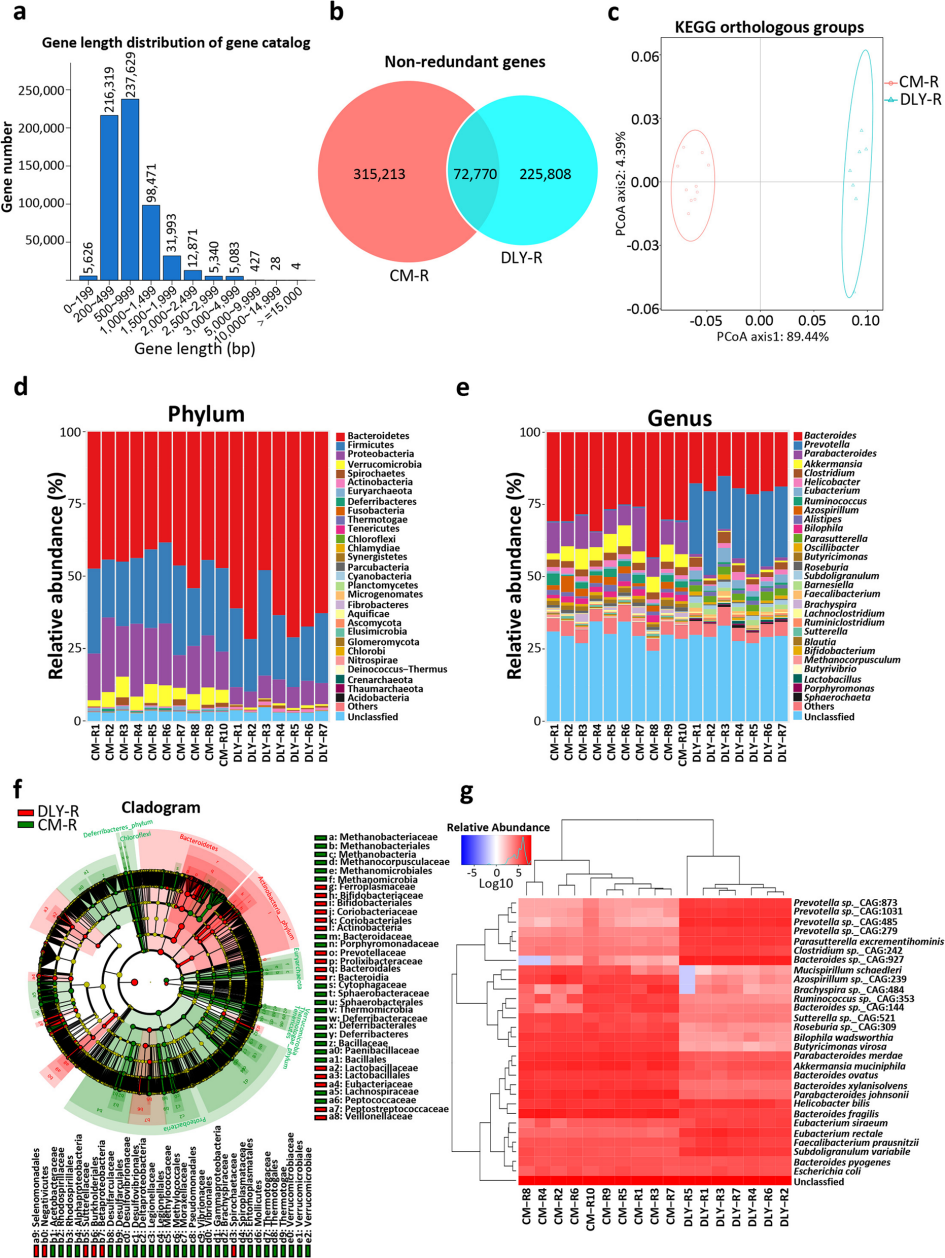
Comparative analysis of the gut microbiome in the recipient germ-free mice treated with fecal microbiota transplantation using metagenomics. **a** Gene length distribution of the gene catalog in the gut microbiome of the recipient GF mice. **b** Venn diagram of gut microbial non-redundant genes. **c** PCoA of gut microbial KOs. **d**, **e** Histogram analysis of microbial taxonomic composition at phylum (**d**) and genus (**e**) levels, respectively. **f** Cladogram of gut microbial composition using LEfSe. **g** Heatmap analysis of microbial taxonomic composition at species level

To identify the specific intestinal microbial species that contribute to the intestinal epithelial barrier, we analyzed the gut microbiome in the recipient GF mice after FMT using metagenomics. Our data identified a total of 613,791 non-redundant (NR) genes, and the gene length mainly ranged from 200 to 1000 bp (Fig. 3a). A total of 72,770 NR genes were shared by the CM-R and DLY-R groups, while 315,213 NR genes were only identified in the CM-R group (Fig. 3b). The PCoA and Circos of KOs showed an obvious difference in the gut microbial functional profiles between the CM-R and DLY-R groups (Fig. 3c and Additional file 4: Fig. S4a). The KEGG pathway analysis indicated that the gut microbiome in the CM-R group was mainly enriched with several KEGG pathways (such as “bacterial chemotaxis”, “flagellar assembly”, “methane metabolism”, “glycosaminoglycan degradation”, “porphyrin and chlorophyll metabolism”, “phosphonate and phosphinate metabolism”, “two-component system”, and “nitrotoluene degradation”) (Additional file 4: Fig. S4b). However, the gut microbiome in the DLY-R group was mainly enriched with several KEGG pathways (such as “alanine, aspartate, and glutamate metabolism”, “lysine biosynthesis”, “protein export”, “the biosynthesis of amino acids”, “phosphotransferase system”, “folate biosynthesis”, and “cyanoamino acid metabolism”) (Additional file 4: Fig. S4b).

Next, we analyzed the gut microbial taxonomic composition in the recipient GF mice. The results of PCoA based on beta diversity showed an obvious difference in the gut microbial composition between the CM-R and DLY-R groups at the phylum (Additional file 5: Fig. S5a) and genus (Additional file 5: Fig. S5b) levels, respectively. The results of the heatmap based on beta diversity further showed an obvious difference in the gut microbial composition between the CM-R and DLY-R groups at the phylum (Additional file 5: Fig. S5c) and genus (Additional file 5: Fig. S5d) levels, respectively. The histogram analysis showed an obvious difference in the gut microbial taxonomic composition between the CM-R and DLY-R groups at the phylum (Fig. 3d) and genus (Fig. 3e) levels, respectively. The results further showed that the relative abundances of the phyla Bacteroidetes and Actinobacteria in CM-R group were lower than those in the DLY-R group (Additional file 6: Fig. S6a). However, the relative abundances of the phyla Proteobacteria and Verrucomicrobia in the CM-R group were higher than those in the DLY-R group (Additional file 6: Fig. S6a). At the genus level, the relative abundances of several genera (such as *Bacteroides*, *Parabacteroides*, and *Akkermansia*) in the CM-R group were higher than those in the DLY-R group (Additional file 6: Fig. S6b). However, the relative abundances of genera *Prevotella* in the CM-R group were lower than those in the DLY-R group (Additional file 6: Fig. S6b).

Further LEfSe analysis indicated that the gut microbiota in the CM-R group was mainly enriched with these families, such as Bacteroidaceae, Porphyromonadaceae, Deferribacteraceae, and Desulfovibrionaceae (Fig. 3f). While the gut microbiota in the DLY-R group was mainly enriched with these families, such as Prevotellaceae (Fig. 3f). The cluster analysis further demonstrated an obvious difference in gut microbial taxonomic composition between the CM-R and DLY-R groups shown in heatmaps at the phylum (Additional file 6: Fig. S6c), genus (Additional file 6: Fig. S6d), and species (Fig. 3g) levels, respectively. Thus, these findings based on metagenomics revealed that both the gut microbial functional profiles and taxonomic compositions were distinct between the recipient GF mice that received the fecal microbiota of CM and DLY pigs, respectively.

### Comparison analysis of gut microbiota in the recipient germ-free mice after fecal microbiota transplantation revealed by 16S rDNA gene amplicon survey

**Fig. 4:**
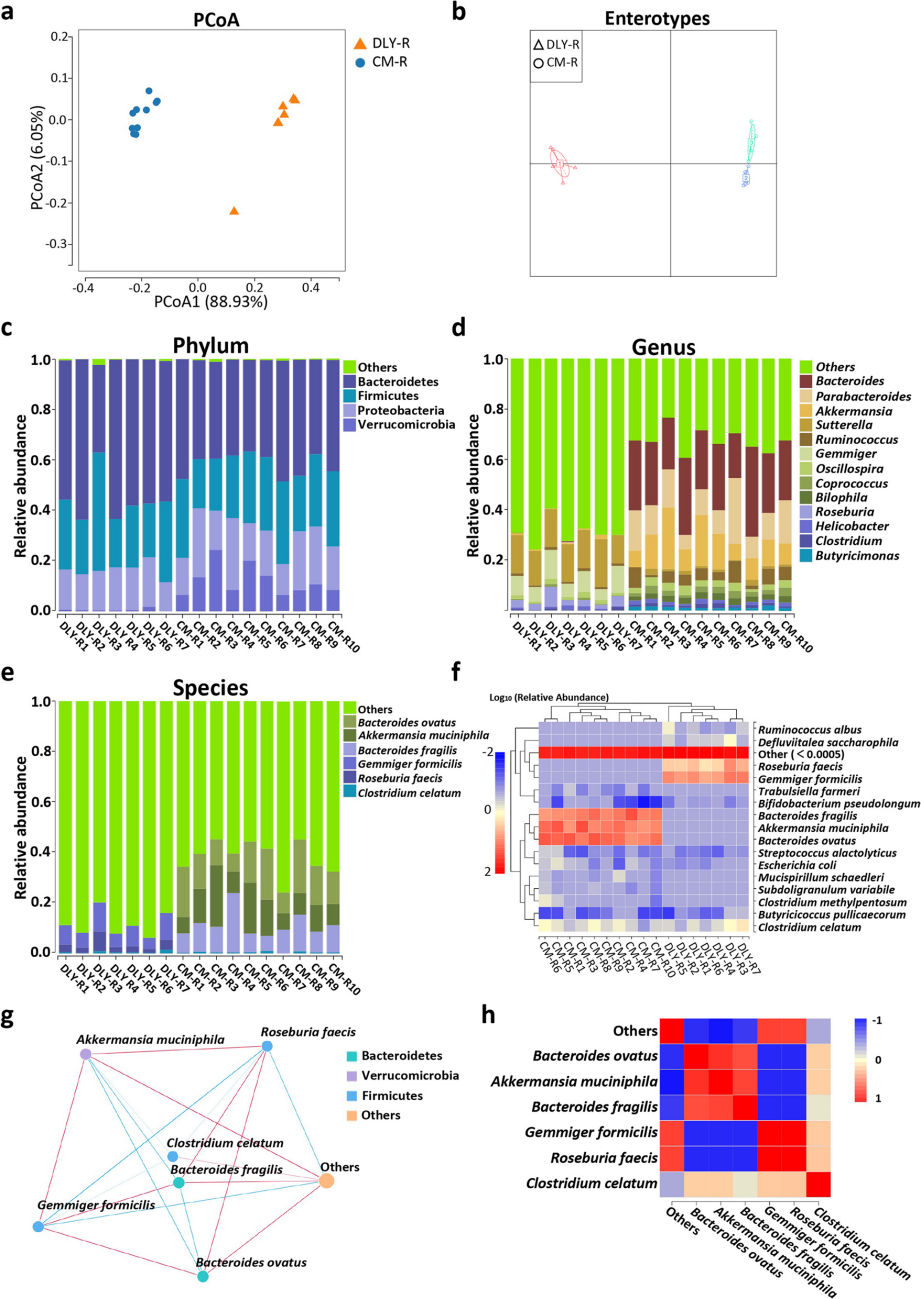
Comparative analysis of gut bacterial communities in the recipient germ-free mice treated with fecal microbiota transplantation by 16S rDNA gene amplicon sequencing. **a** PCoA of gut bacterial beta diversity based on weighted Unifrac distance. **b** Gut bacterial enterotypes analysis. **c**–**e** Histogram analysis of gut bacterial taxonomic composition at phylum (**c**), genus (**d**), and species (**e**) levels, respectively. **f** Heatmap analysis of gut bacterial taxonomic composition at species level. **g** Cytoscape analysis of gut bacterial species. **h** Heatmap analysis of gut bacterial species based on Spearman’s correlation

Previous studies have suggested that gut bacterial community results from 16S rDNA amplicon sequencing and metagenomics may be distinct [32, 33]. We further utilized a 16S rDNA gene amplicon sequencing to analyze the gut bacterial community in the recipient GF mice after FMT. Our sequencing data were sufficient to capture almost all the bacterial species shown in the rarefaction curves (Additional file 7: Fig. S7a). A total of 14 ASVs were identified in both the DLY-R and CM-R groups, while 171 ASVs were only identified in the CM-R group (Additional file 7: Fig. S7b). The PCoA showed an obvious difference in beta diversity between the DLY-R and CM-R groups (Fig. 4a). Cluster analysis further suggested an obvious difference in beta diversity between the DLY-R and CM-R groups in the cluster tree (Additional file 7: Fig. S7c) and heatmap (Additional file 7: Fig. S7d). Our results showed that the gut bacterial community in CM-R group exhibited a trend of higher alpha diversity than that in the DLY-R group, as evidenced by the Chao (Additional file 7: Fig. S7e) and Shannon indices (Additional file 7: Fig. S7f). The enterotype analysis identified that the CM-R and DLY-R groups belonged to different enterotypes, suggesting a distinct bacterial composition (Fig. 4b). The GraPhlAn analysis revealed that the main phyla included Bacteroidetes, Firmicutes, Proteobacteria, and Verrucomicrobia. The main genera included *Bacteroides* belonging to family Bacteroidaceae, *Parabacteroides* belonging to family Porphyromonadaceae, *Sutterella* belonging to family Alcaligenaceae, and *Akkermansia* belonging to family Verrucomicrobiaceae in the recipient GF mice (Additional file 7: Fig. S7g). The LEfSe analysis revealed that the gut bacterial communities in the CM-R group were especially enriched with bacteria belonging to the families, such as Bacteroidaceae, Porphyromonadaceae, Deferribacteraceae, Verrucomicrobiaceae, and Desulfovibrionaceae (Additional file 7: Fig. S7h).

The histogram analysis of taxonomic compositions further showed that several phyla (including Bacteroidetes, Firmicutes, Proteobacteria, and Verrucomicrobia) were predominant in the gut of the recipient GF mice (Fig. 4c). The relative abundance of the phylum Bacteroidetes in CM-R group was lower than those in the DLY-R group, while the relative abundance of the phylum Verrucomicrobia in the CM-R group were higher than those in the DLY-R group (Additional file 8: Fig. S8a). Several genera including *Bacteroides*, *Parabacteroides*, *Akkermansia*, *Sutterella*, *Ruminococcus*, *Gemmiger*, *Oscillospira*, *Coprococcus*, *Bilophila*, *Roseburia*, *Helicobacter*, *Clostridium*, and *Butyricimonas* were predominant in the gut of the recipient GF mice (Fig. 4d). The relative abundance of several genera (such as *Bacteroides*, *Parabacteroides*, *Akkermansia*, and *Ruminococcus*) in the CM-R group were higher than those in the DLY-R group (Additional file 8: Fig. S8b). Several species including *Bacteroides ovatus*, *Akkermansia muciniphila*, *Bacteroides fragilis*, *Gemmiger formicilis*, *Roseburia faecis*, and *Clostridium celatum* were the gut predominant species in the recipient GF mice (Fig. 4e). The cluster analysis indicated that the CM-R group was especially enriched with three species (*B. fragilis*, *A. muciniphila*, and *B. ovatus*), as is shown in the heatmap (Fig. 4f). Our results indicated that there were strong correlations among six bacterial species (*B. ovatus*, *A. muciniphila*, *B. fragilis*, *G. formicilis*, *R. faecis*, and *C. celatum*), as evidenced by the network based on Cytoscape (Fig. 4g) and the heatmap based on the Spearman correlation (Fig. 4h). We further used the PICRUSt2 to predict the gut bacterial functional profiles. The results showed that several KEGG pathways (such as “transport and catabolism”, “cell motility”, “immune system”, “digestive system”, “metabolism of cofactors and vitamins”, and “glycan biosynthesis and metabolism”) were especially enriched in CM-R group (Additional file 9: Fig. S9a). Several COG functions (such as “lipid transport and metabolism”, “secondary metabolites biosynthesis, transport, and catabolism”, and “coenzyme transport and metabolism”) were especially enriched in CM-R group (Additional file 9: Fig. S9b). Overall, these findings based on the 16S rDNA gene amplicon survey revealed that the gut bacterial diversity and taxonomic composition were distinct between the recipient GF mice that received the fecal microbiota of CM and DLY pigs, respectively.

We also compared the gut bacterial communities between the donor pigs and the recipient GF mice. The results of PCoA showed that CM-R group was relatively close to CM-D group (donor CM pigs) in the beta diversity (Additional file 10: Fig. S10a). The results of PCoA also showed that DLY-R group was relatively close to DLY-D group (donor DLY pigs) in the beta diversity (Additional file 10: Fig. S10a). The heatmap analysis further showed CM-D group was clustered together with CM-R group, suggesting that the gut bacterial genus-level taxonomic composition was similar between CM-D and CM-R groups (Additional file 10: Fig. S10b). The heatmap analysis showed DLY-D group was clustered together with DLY-R group, suggesting that the gut bacterial genus-level taxonomic composition was similar between DLY-D and DLY-R groups (Additional file 10: Fig. S10b). Thus, these findings suggested an efficacy of FMT that transferred the gut bacterial community characteristics from pigs to GF mice.

### Oral administration of *Bacteroides fragilis* promotes intestinal epithelial barrier function

**Fig. 5:**
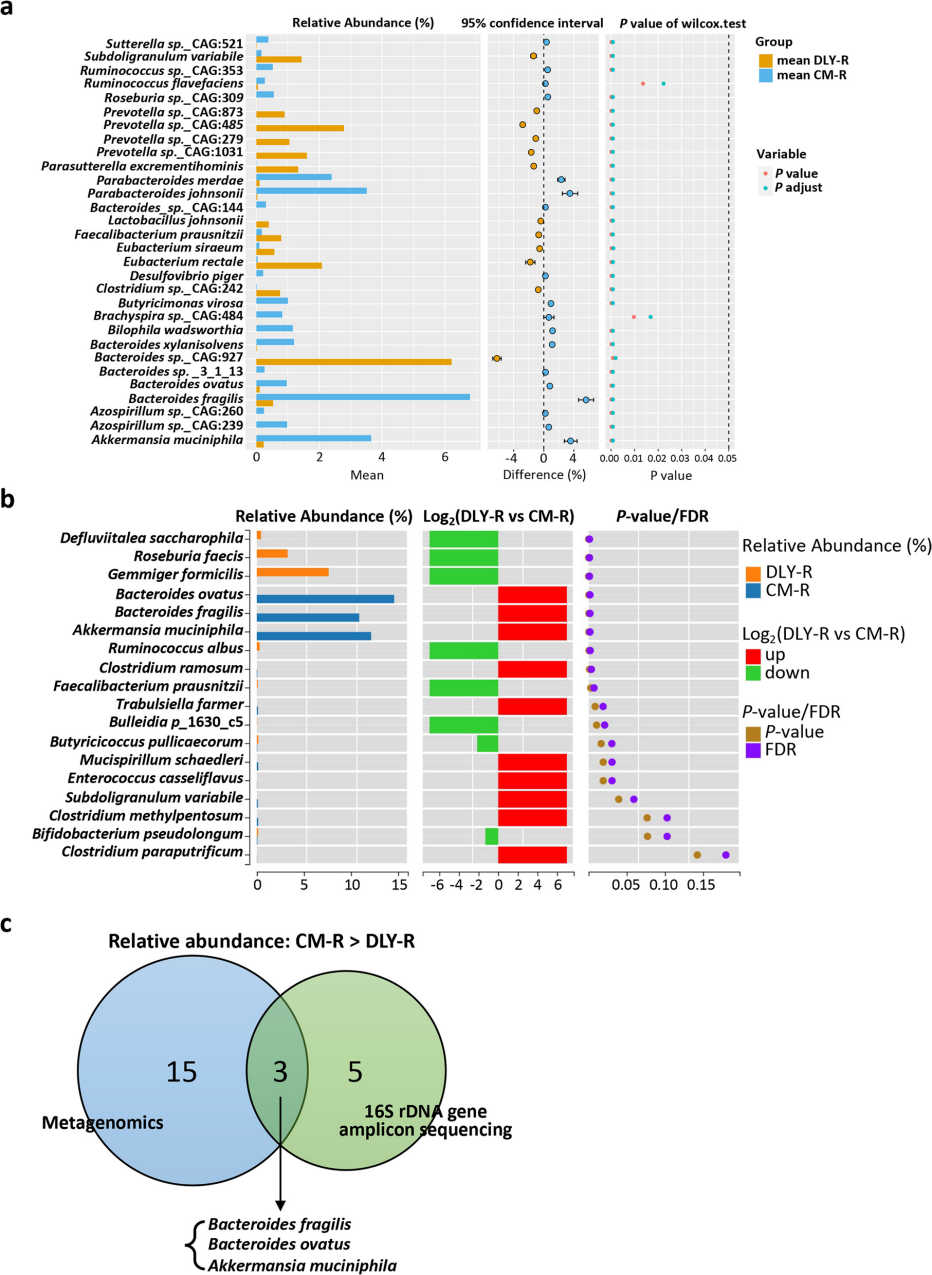
Screening of gut microbial species candidates that contribute to intestinal epithelial barrier function. **a** Comparison analysis of the relative abundances of gut microbial species by metagenomics. **b** Comparison analysis of the relative abundances of gut bacterial species by 16S rDNA gene amplicon survey. **c** Venn diagram analysis of gut microbial species. The numbers of the corresponding microbial species whose relative abundances in CM-R group are higher than those in DLY-R group are shown in Venn diagram. The differential microbial species were identified by metagenomics and 16S rDNA gene amplicon survey, respectively. The data are presented as the mean relative abundance and evaluated using the Wilcoxon rank-sum test; *n* = 10 (CM-R group) and *n* = 7 (DLY-R group)

Next, we screened for potential gut microbial species that may contribute to the intestinal epithelial barrier. We used the two methods (including 16S rDNA amplicon sequencing and metagenomics) to obtain more precise results of differentially bacterial species between CM-R group and DLY-R group. The metagenomics analysis showed that the relative abundance of 18 microbial species in the CM-R group were higher than those in the DLY-R group, and all of them were bacterial species (Fig. 5a). The 16S rDNA gene amplicon sequencing analysis indicated that the relative abundances of eight bacterial species in the CM-R group were higher than those in the DLY-R group (Fig. 5b). Furthermore, a total of three bacterial species (*B. ovatus*, *B. fragilis*, and *A. muciniphila*) were identified as the common species whose relative abundances were higher in the CM-R group than those in the DLY-R group as evidenced by both the metagenomics and 16S rDNA gene amplicon survey (Fig. 5c). Therefore, we determined that these three bacterial species were positively correlated with intestinal epithelial barrier function.

**Fig. 6:**
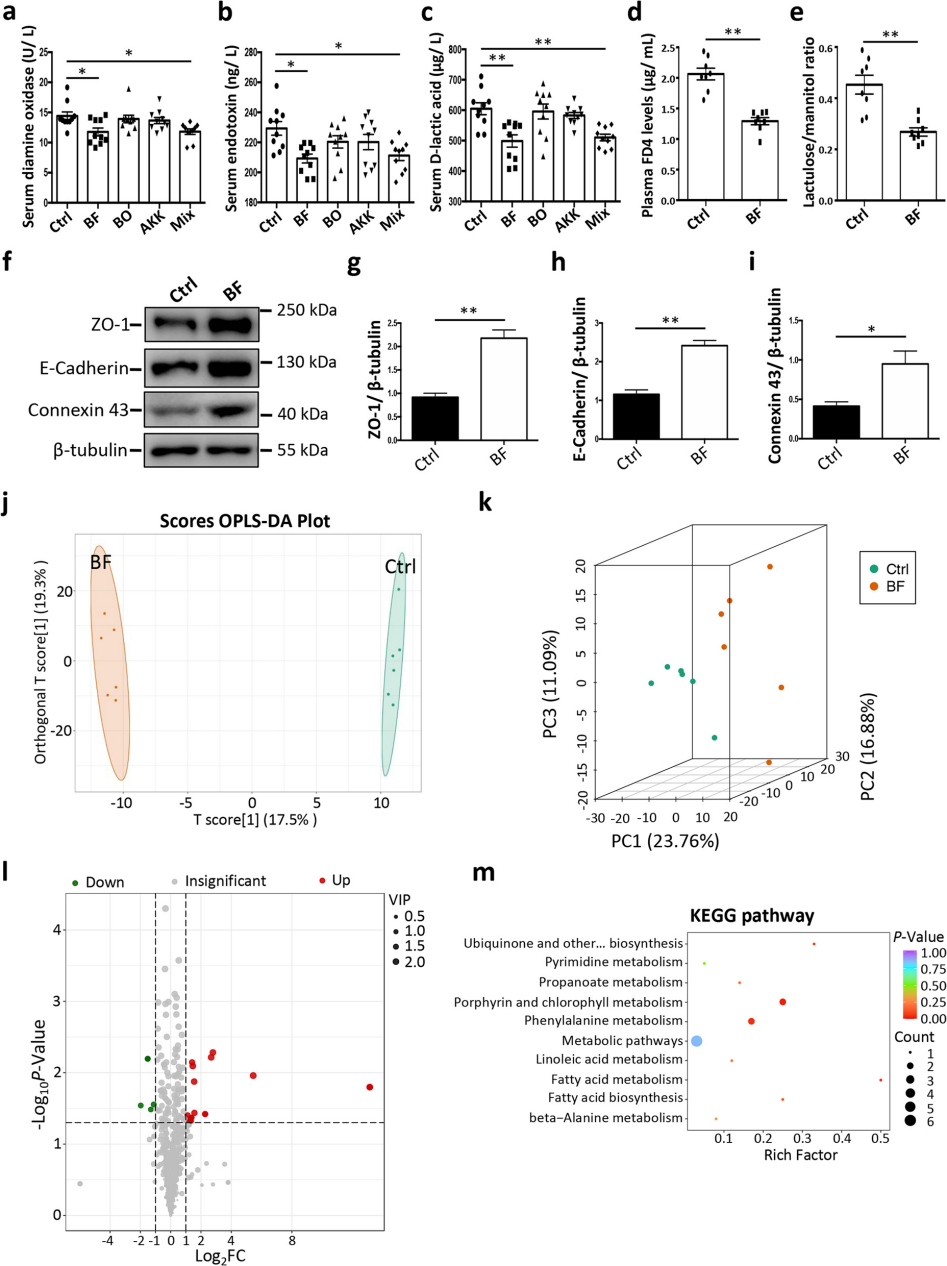
The oral administration of *Bacteroides fragilis* facilitates the intestinal epithelial barrier function and serum metabolomics analysis. **a** Activities of serum diamine oxidase (Ctrl control, BF *Bacteroides fragilis*, BO *Bacteroides ovatus*, AKK *Akkermansia muciniphila*; Mix, three-bacteria consortium [containing equal numbers of *Bacteroides fragilis*, *Bacteroides ovatus*, and *Akkermansia muciniphila*]). **b** Levels of serum endotoxin. **c** Levels of serum D-lactic acid. **d** Plasma FD4 levels. **e** Lactulose/mannitol ratio. **f** Representative western blotting of ZO-1, E-cadherin, Connexin 43, and β-tubulin in jejunal epithelium of mice. **g**–**i** Quantitation of the ZO-1 (**g**), E-cadherin (**h**), Connexin 43 (**i**) levels normalized to β-tubulin levels. **j** OPLS-DA of serum metabolites. **k** Principal component analysis of serum metabolites. **l** Volcano plot analysis of serum metabolites. **m** The enrichment analysis of KEGG pathways by differential serum metabolites. The data are presented as the mean ± SEM and evaluated by one-way ANOVA with adjustment for multiple comparisons in **a**–**c** (*n* = 10). The data are presented as the mean ± SEM and evaluated using Student’s *t*-test in **d** (*n* = 8), **e** (*n* = 8), and **g**–**i** (*n* = 3). In addition, the data were assessed by the VIP ≥ 1 from OPLS-DA, absolute Log_2_ (fold change) ≥ 1, and *p*-value < 0.05 in **l** (*n* = 6). **p < 0.01, *p < 0.05

We transferred the three bacterial suspensions (including *B. ovatus*, *B. fragilis*, and *A. muciniphila*) to mice to investigate their potential roles in intestinal epithelial barrier function. Our data indicated that oral gavage of *B. fragilis* alone or a consortium of three bacterial species (including *B. ovatus*, *B. fragilis*, and *A. muciniphila*) significantly decreased the serum diamine oxidase activities, endotoxin levels, and D-lactic acid levels, thereby suggesting an enhanced intestinal epithelial barrier function (Fig. 6a–c). The data showed that FD4 levels in plasma and lactulose/mannitol ratio were significantly decreased by *B. fragilis* treatment (Fig. 6d, e). Our results demonstrated that the oral administration of *B. fragilis* significantly increased the levels of jejunal epithelial ZO-1, E-cadherin, and Connexin 43 (Fig. 6f–i). The results of immuohistochemical staining showed that the localization of jejunal epithelial ZO-1, E-cadherin, and Connexin 43, was not altered by oral administration of *B. fragilis* (Additional file 11: Fig. S11). Our data also demonstrated that jejunal epithelial RegIIIγ levels were not significantly changed by oral administration of *B. fragilis* (Additional file 12: Fig. S12). The results of negative controls did not show obvious staining intensity, suggesting the specific roles of primary antibodies (Additional files 11 and 12: Figs. S11 and 12). The intestinal morphology analysis also showed that neither the villus height nor the crypt depth was significantly changed by the oral administration of *B. fragilis* (Additional file 13: Fig. S13). These results reveal the beneficial role of *B. fragilis* in intestinal epithelial barrier function.

### *Bacteroides fragilis*-derived 3-phenylpropionic acid metabolite contributes to intestinal epithelial barrier function

Next, we investigated the mechanism underlying of *B. fragilis*-mediated intestinal epithelial barrier function. Growing evidence has suggested that gut microbial metabolites may be the intermediates for host-gut microbiota interaction [34]. Thus, we performed a metabolomic analysis to identify the serum metabolite candidates involved in the *B. fragilis*-mediated intestinal epithelial barrier function. The OPLS-DA (Fig. 6j) and PCA (Fig. 6k) indicated an obvious difference in the serum metabolite composition between the control group and the *B. fragilis* group, thereby suggesting a potent effect of *B. fragilis* on host physiology. The volcano plot further showed the differential serum metabolites affected by *B. fragilis* (Fig. 6l). The results showed that a total of 18 serum metabolites were upregulated, while 5 serum metabolites were downregulated by the oral gavage of *B. fragilis* (Fig. 6l). Several KEGG pathways (such as “porphyrin and chlorophyll metabolism”, “phenylalanine metabolism”, and “fatty acid metabolism”) were enriched with the differential serum metabolites (Fig. 6m).

**Fig. 7:**
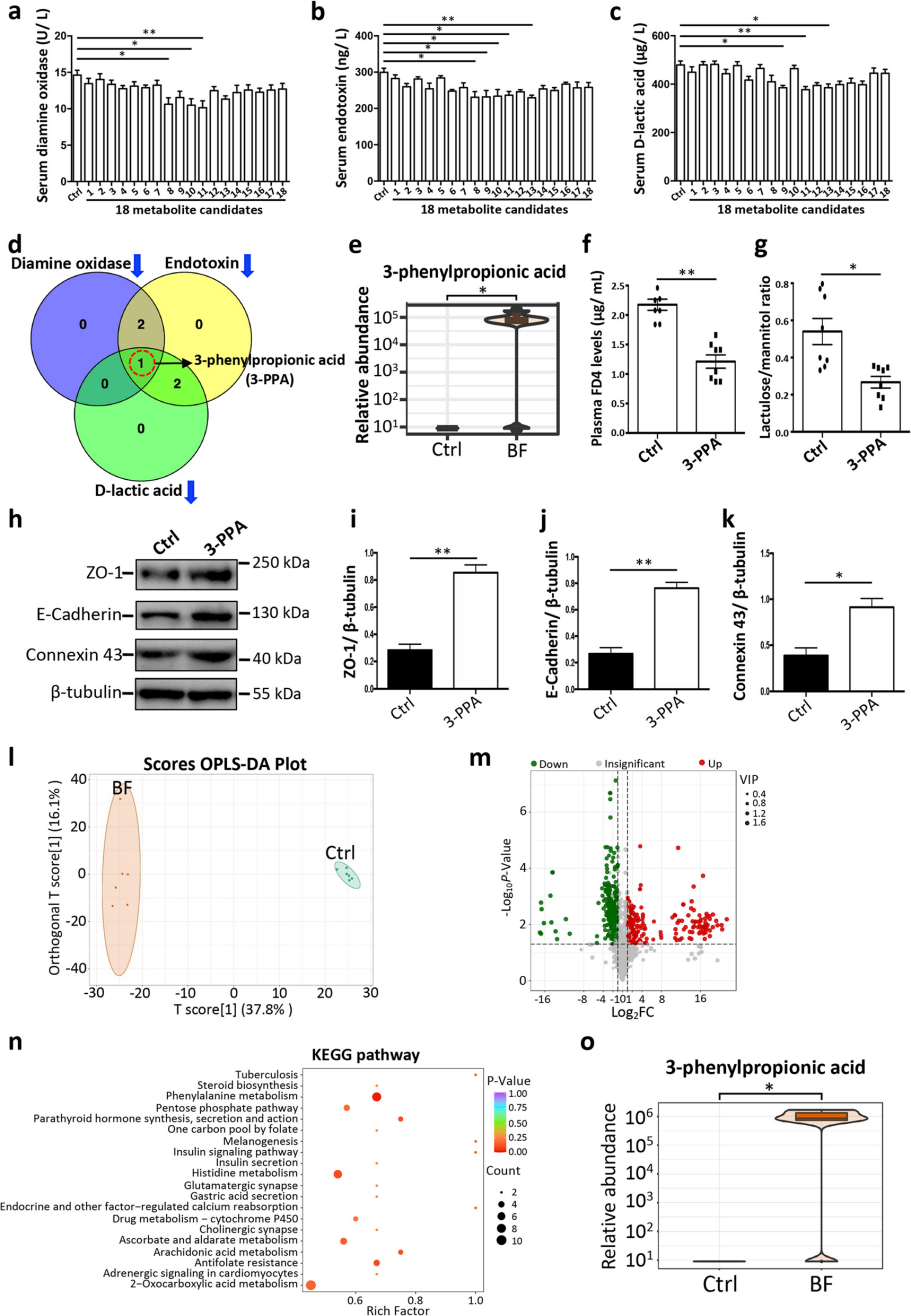
*Bacteroides fragilis*-derived 3-phenylpropionic acid confers the beneficial effects on the intestinal epithelial barrier function. **a** Activities of serum diamine oxidase (Ctrl control; 1 to 18, the first metabolite candidate to the 18th metabolite candidate and the detailed metabolite names were shown in the Methods section). **b** Levels of serum endotoxin. **c** Levels of serum D-lactic acid. **d** Venn diagram analysis of metabolite candidates that decrease the serum diamine oxidase activities, endotoxin levels, and D-lactic acid levels, respectively. The numbers of corresponding metabolite candidates were shown in the Venn diagram. **e** Relative abundance analysis of serum 3-phenylpropionic acid in mice (Ctrl control, BF *Bacteroides fragilis*). **f** Plasma FD4 levels (3-phenylpropionic acid, 3-PPA). **g** Lactulose/mannitol ratio. **h** Representative western blotting of ZO-1, E-cadherin, Connexin 43, and β-tubulin in the jejunal epithelium of mice. **i**–**k** Quantitation of the ZO-1 (**i**), E-cadherin (**j**), and Connexin 43 (**k**) levels normalized to β-tubulin levels. **l** The OPLS-DA of fecal metabolites. **m** The volcano plot analysis of fecal metabolites. **n** The enrichment analysis of KEGG pathways by differential fecal metabolites. **o** Relative abundance analysis of fecal 3-phenylpropionic acid in mice. The data are presented as the mean ± SEM and evaluated by one-way ANOVA with adjustment for multiple comparisons in **a**–**c** (*n* = 5). The data are presented as the mean ± SEM and evaluated by Student’s *t*-test in **f** (*n* = 8), **g** (*n* = 8), and **i**–**k** (*n* = 3). In addition, the data were assessed by the VIP ≥ 1 from OPLS-DA, absolute Log_2_ (fold change) ≥ 1, and *p*-value < 0.05 in **m** (*n* = 6). **p < 0.01, *p < 0.05

We then transferred these 18 serum metabolites, which were upregulated by the *B. fragilis*, into mice by oral gavage and evaluated the intestinal epithelial barrier function of mice. The results demonstrated that serum diamine oxidase activities of mice were decreased significantly with the treatments including three metabolites (N-acetyl-L-glutamine, 2-hydroxybutanoic acid, and 3-phenylpropionic acid) (Fig. 7a). The serum endotoxin levels in mice were decreased significantly with the treatments including five metabolites (N-acetyl-l-glutamine, L-fucose, 2-hydroxybutanoic acid, 3-phenylpropionic acid, and L-rhamnose) (Fig. 7b). The serum D-lactic acid levels in mice were decreased significantly with the treatments including three metabolites (L-fucose, 3-phenylpropionic acid, and L-rhamnose) (Fig. 7c). Interestingly, the Venn diagram showed that only one metabolite, 3-phenylpropionic acid, significantly decreased all the indicators (including serum diamine oxidase activities, endotoxin levels, and D-lactic acid levels) (Fig. 7d). The metabolomic analysis showed that 3-phenylpropionic acid was the serum metabolite with the highest fold of change among all the serum metabolites upregulated by *B. fragilis* (Figs. 6l and 7e). The results showed that the FD4 levels in plasma and lactulose/mannitol ratio were significantly decreased by 3-phenylpropionic acid treatment (Fig. 7f, g). Our results further demonstrated that the levels of intestinal epithelial ZO-1, E-cadherin, and Connexin 43 were significantly increased by 3-phenylpropionic acid (Fig. 7h–k). These findings indicated that 3-phenylpropionic acid confers the beneficial effects on the intestinal epithelial barrier function.

We further performed the metabolomic analysis to identify fecal metabolites in mice affected by *B. fragilis*. The results of OPLS-DA showed an obvious difference in the fecal metabolite compositions between the control and *B. fragilis* groups (Fig. 7l). The volcano plot further showed that 252 fecal metabolites were upregulated, while 331 fecal metabolites were downregulated by the oral gavage of *B. fragilis* (Fig. 7m). Several KEGG pathways (such as “phenylalanine metabolism”, “histidine metabolism”, and “ascorbate and aldarate metabolism”) were enriched with the differential fecal metabolites (Fig. 7n). Interestingly, 3-phenylpropionic acid in feces of mice was also significantly increased with the treatment of *B. fragilis* (Fig. 7o), suggesting that 3-phenylpropionic acid may be produced by intestinal *B. fragilis* and then absorbed into the blood. Together, these findings demonstrated that 3-phenylpropionic acid, a key metabolite derived from *B. fragilis*, contributes to the intestinal epithelial barrier function.

### 3-Phenylpropionic acid facilitates intestinal epithelial barrier function through AhR signaling

**Fig. 8:**
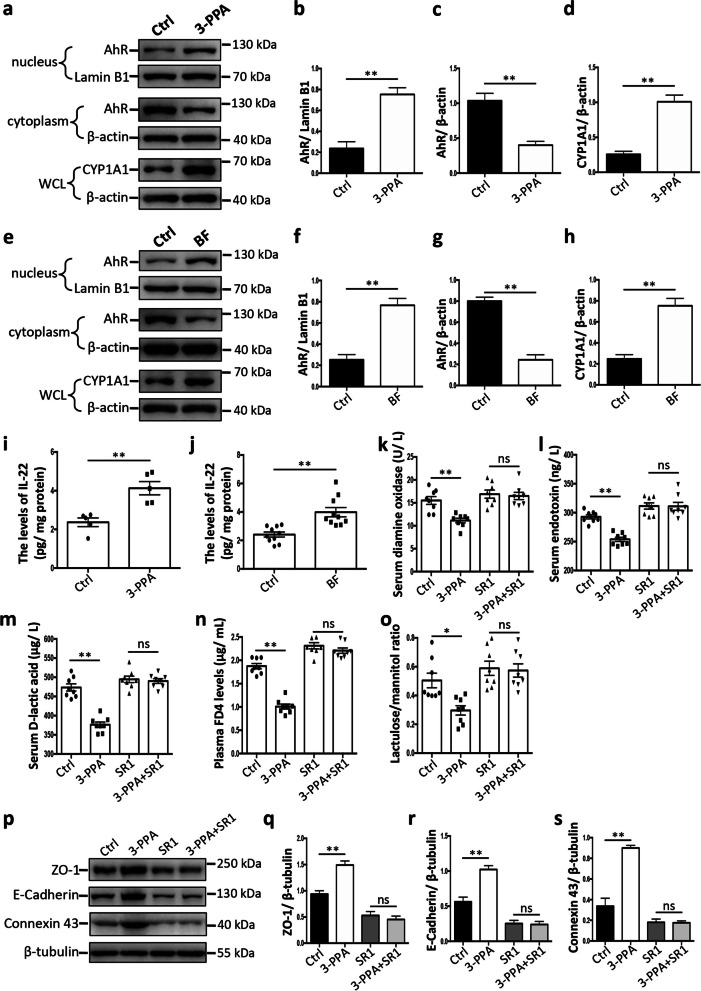
AhR signaling is essential for the 3-phenylpropionic acid-mediated enhancement on the intestinal epithelial barrier function. **a** Representative western blotting of AhR, Lamin B1, β-actin, and CYP1A1 in the jejunal epithelium of mice from the control (Ctrl) and 3-phenylpropionic acid (3-PPA) groups. **b**–**d** Quantitation of AhR levels (**b**) normalized to Lamin B1 levels. Quantitation of AhR (**c**) and CYP1A1 (**d**) levels normalized to β-actin levels. **e** Representative western blotting of AhR, Lamin B1, β-actin, and CYP1A1 in the jejunal epithelium of mice from the Ctrl and *Bacteroides fragilis* (BF) groups. **f**–**h** Quantitation of AhR levels (**f**) normalized to Lamin B1 levels. Quantitation of AhR (**g**) and CYP1A1 (**h**) levels normalized to β-actin levels. **i**, **j** Levels of IL-22 in the jejunal epithelium of mice. **k**–**m** Activities of serum diamine oxidase (**k**) and the levels of endotoxin (**l**) and D-lactic acid (**m**), respectively (SR1 StemRegenin 1). **n** Plasma FD4 levels. **o** Lactulose/mannitol ratio. **p** Representative western blotting of ZO-1, E-cadherin, Connexin 43, and β-tubulin in jejunal epithelium of mice. **q**–**s** Quantitation of the ZO-1 (**q**), E-cadherin (**r**), and Connexin 43 (**s**) levels normalized to β-tubulin levels. The data are presented as the mean ± SEM and evaluated by Student’s *t*-test in **b**–**d** (*n* = 3), **f**–**h** (*n* = 3), **i** (*n* = 5), and **j** (*n* = 10). The data are presented as the mean ± SEM and evaluated by one-way ANOVA with adjustment for multiple comparisons in **k**–**o** (*n* = 8) and **q**–**s** (*n* = 3). **p < 0.01, *p < 0.05; ns not significant

Next, we investigated the mechanism underlying 3-phenylpropionic acid-mediated intestinal epithelial barrier function. Given that AhR signaling may be involved in the regulatory roles of aryl hydrocarbon derivatives [35], we examined whether the AhR signaling was activated by 3-phenylpropionic acid. The results demonstrated that the translocation of AhR from the cytoplasm into the nucleus was promoted by the 3-phenylpropionic acid treatment (Fig. 8a–d). CYP1A1, a protein downstream of AhR signaling, was significantly upregulated by 3-phenylpropionic acid (Fig. 8a–d). Our results also demonstrated that the translocation of AhR from the cytoplasm into the nucleus was promoted and that CYP1A1 was significantly upregulated by the *B. fragilis* treatment (Fig. 8e–h). Our results further showed that IL-22, which is also downstream of the AhR signaling, was significantly upregulated by both the 3-phenylpropionic acid and *B. fragilis* treatment (Fig. 8i, j). These results revealed that AhR signaling was activated by *B. fragilis*-derived 3-phenylpropionic acid.

We then evaluated whether AhR signaling is essential for 3-phenylpropionic acid-mediated intestinal epithelial barrier function. The results showed that StemRegenin 1, an AhR inhibitor, blocked the roles of 3-phenylpropionic acid in AhR signaling activation (Additional file 14: Fig. S14). Our results demonstrated that the oral gavage of StemRegenin 1 inhibited the regulatory roles of 3-phenylpropionic acid in serum diamine oxidase activities, endotoxin levels, and D-lactic acid levels (Fig. 8k–m). The results showed that StemRegenin 1 inhibited the regulatory roles of 3-phenylpropionic acid in the FD4 levels in plasma and lactulose/mannitol ratio (Fig. 8n, o). StemRegenin 1 also inhibited the promoting roles of 3-phenylpropionic acid on the intestinal epithelial levels of ZO-1, E-cadherin, and Connexin 43 (Fig. 8p–s). Together, these findings revealed that *B. fragilis*-derived 3-phenylpropionic acid promotes intestinal epithelial barrier function through AhR signaling.

## Discussion

Given that the intestinal epithelial barrier disorder is associated with several gastrointestinal diseases (such as IBD and IBS) and has detrimental effects on host homeostasis maintenance [1, 3], preventive strategies are urgently required. Here, we screened and validated *B. fragilis* as a bacterial species that confers beneficial roles in the intestinal epithelial barrier function, by combining the FMT, metagenomics, and 16S rDNA gene amplicon sequencing. First, we studied the gut microbiome of seven pig breeds and found an obvious difference in the gut microbiome between native CM pigs and commercial crossbred DLY pigs. CM pigs had a stronger intestinal epithelial barrier function than DLY pigs. Next, we demonstrated that FMT from two pig breeds to GF mice transferred the intestinal epithelial barrier characteristics. Then, we screened the bacterial candidates that mediated intestinal epithelial barrier function. Our previous study also revealed that FMT from native CM piglets to commercial crossbred LY piglets deceased the incidence of diarrhea in early-weaned piglets and identified two key microbial species that mediate the diarrhea resistance [17]. These results suggest that native breeds of livestock confer potential intestinal microbial resources that may be associated with the higher disease resistance and stress tolerance than commercial breeds.

Precise probiotic manipulation is increasingly being recognized as an important strategy for preventing gastrointestinal dysfunction [9], although many studies have demonstrated the efficacy of FMT [36]. Recent evidence suggests that FMT may increase the risk of transmitting pathogens to recipients [37, 38]. Several factors (such as donor selection, fecal material transfer, and recipient parameters) may also affect the efficacy of FMT [39]. Thus, mining the functional gut microbes that mediate host health is urgently needed. Interestingly, FMT may contribute to establishing the potential relationships between gut microbiota and host characteristics (such as obesity and regulatory T cells induction) [40, 41]. Growing evidence has shown that gut microbial omics (such as metagenomics, bacterial 16S rDNA gene amplicon sequencing, and fungal ITS gene amplicon sequencing) help to further identify the specific gut microbial species that mediate host health, such as colitis [42], obesity [43], *Clostridium difficile* infection resistance [44], and antitumor immunity [45]. To specifically screen the gut microbial species candidates, we combined the metagenomics and bacterial 16S rDNA gene amplicon sequencing to dissect the difference in the gut microbiota between the recipient GF mice in this study. Therefore, the use of gut microbial omics will provide a technical basis for the high-throughput screening of functional microbial species and thus contributes to the precise probiotics manipulation in preventing gastrointestinal dysfunction.

Many studies have demonstrated that GF mice constitute an appropriate research model for the investigation of gut microbiota-host interactions [46]. A previous study indicated that gut microbiota has a critical role in host fat deposition by transferring the fecal microbiota from obese and lean humans to GF mice, respectively [41]. A recent study revealed that FMT from patients with colorectal cancer and healthy individuals to GF mice transferred the intestinal carcinogenesis characteristics, suggesting a relationship between gut microbial alterations and the development of colorectal cancer [47]. Considering that host genetics has important impacts on physiological metabolism, we also used a GF mouse model to eliminate the influence of host genetics. In the present study, FMT from native CM pigs and commercial DLY pigs to GF mice transferred the intestinal epithelial barrier characteristics, suggesting that gut microbiota has a vital function on intestinal epithelial barrier regulation. Our data indicated that compared to DLY-R group, the gut microbial communities in the recipient GF mice, that received the fecal microbiota from native CM finishing pigs, were mainly enriched with these families, such as Bacteroidaceae, Porphyromonadaceae, and Desulfovibrionaceae. Interestingly, gut microbial communities in native CM finishing pigs were also mainly enriched with these families, such as Bacteroidaceae, Porphyromonadaceae, and Desulfovibrionaceae, compared to commercial DLY finishing pigs. These findings suggested an efficacy of FMT using GF mice as the recipients. Therefore, GF animals are increasingly being used as important models for the evaluation of the function of gut microbiota in host physiology. A previous study showed that the gut permeability in GF mice increased with FMT treatment using one healthy adult human donor, and the gut permeability reached the highest levels within a week [48]. Our data revealed that the GF mice in CM-R group had a lower gut permeability than those in DLY-R group after FMT treatment every 3 days for 5 weeks. The FMT treatment period of 5 weeks in our study was longer than the period of 1 week in the previous study. Our results also demonstrated that the gut length index, the intestinal morphology, and the number of intestinal goblet cells were not significantly different between CM-R and DLY-R groups. Thus, compared to DLY-R group, the lower permeability observed in CM-R group may not reflect a default in maturation of the gut with the CM microbiota. The difference in the gut permeability shown in our data may mainly be the result of the stimulation of different microbiota for a longer time than that in the previous study.

Mining the gut functional microbial species that enhance intestinal barrier function is still urgently needed, although previous studies have demonstrated the vital function of gut microbiota in intestinal epithelial barrier maintenance [15, 49]. Our results identified *B. fragilis* as a microbial species that promotes intestinal epithelial barrier function, suggesting that it may be a potential probiotic for intestinal health maintenance. Gut microbiota promotes intestinal epithelial barrier function mainly via microbial structural components and metabolites [15]. Following the high-throughput screening of metabolites via metabolomics, we further demonstrated that *B. fragilis*-derived 3-phenylpropionic acid plays beneficial roles in intestinal epithelial barrier function. Furthermore, we showed that 3-phenylpropionic acid can activate the intestinal epithelial AhR signaling and that *B. fragilis*-derived 3-phenylpropionic acid enhanced the intestinal epithelial barrier through AhR signaling. These data further supported that aryl hydrocarbon derivatives have the potential to activate the AhR signaling and the regulatory roles of aryl hydrocarbon derivatives may be involved with AhR signaling. Thus, our findings reveal a mechanism of gut microbiota-driven intestinal epithelial barrier function and further suggest these important targets for protection against intestinal disorders. These results will further deepen our understandings of that gut microbial metabolites may be the intermediates for host-gut microbiota interaction.

## Conclusions

In sum, we identified *B. fragilis* as a bacterial species that contributes to intestinal epithelial barrier function, using FMT, metagenomics, and 16S rDNA gene amplicon sequencing analysis. Our findings indicate that *B. fragilis*-derived 3-phenylpropionic acid activates AhR signaling to confer the beneficial effects on the intestinal epithelial barrier. These results suggest that the gut microbes that produce 3-phenylpropionic acid may also have regulatory roles in the intestinal epithelial barrier and that 3-phenylpropionic acid may be a potential biomarker for intestinal epithelial barrier maintenance. Our findings suggest a potential avenue to prevent intestinal barrier dysfunction in mammals.

## Supplementary Information

**Additional file 1: Fig. S1.** Analyses of blood routine indices and organ indices in GF mice treated with FMT. (**a**-**g**) The number of peripheral white blood cells (**a**), lymphocytes (**b**), monocytes (**c**), neutrophils (**d**), red blood cells (**e**), and blood platelets (**f**) in mice and the levels of peripheral hemoglobin (**g**) in mice (CM-R, the recipient GF mice that received the fecal microbiota from Congjiang miniature pigs; DLY-R, the recipient GF mice that received the fecal microbiota from Duroc × [Landrace × Yorkshire] pigs). (**h**-**n**) The heart (**h**), liver (**i**), spleen (**j**), kidney (**k**), thymus (**l**), epididymal fat (**m**), and gut length indices (**n**) in mice. The data are presented as the mean ± SEM and evaluated using Student's *t*-test; n = 10 (CM-R) and n = 7 (DLY-R). **p < 0.01; ns, not significant.

**Additional file 2: Fig. S2.** Analysis of intestinal histological morphology in GF mice treated with FMT. (**a**) Representative images of intestinal histological morphology by hematoxylin and eosin staining of duodenum, jejunum, and ileum, respectively. (**b**-**d**) Statistical analysis of the villus height (**b**), crypt depth (**c**), and the ratio of the villus height to the crypt depth (**d**). The data are presented as mean ± SEM and evaluated by two-way analysis of variance (ANOVA); n = 10 (CM-R) and n = 7 (DLY-R); ns, not significant.

**Additional file 3: Fig. S3.** Analysis of the numbers of intestinal goblet cells in GF mice treated with FMT. (**a**) Representative images of intestinal goblet cells stained with PAS staining of the duodenum, jejunum, and ileum, respectively. (**b**) Statistical analysis of goblet cell numbers in the duodenum, jejunum, and ileum, respectively. The data are presented as the mean ± SEM and evaluated by two-way ANOVA; n = 5; ns, not significant.

**Additional file 4: Fig. S4.** Analysis of gut microbial functional profiles in GF mice treated with FMT by metagenomics. (**a**) Circos analysis of gut microbial KEGG orthologous groups (KOs). (**b**) Enrichment analysis of KEGG pathways in the gut microbiome.

**Additional file 5: Fig. S5.** Analysis of gut microbial taxonomic composition based on beta diversity by metagenomics. (**a** and **b**) PCoA of gut microbial taxonomic composition based on beta diversity at phylum (**a**) and genus (**b**) levels, respectively. (**c** and **d**) Heatmap analysis of gut taxonomic composition based on beta diversity at phylum (**c**) and genus (**d**) levels, respectively.

**Additional file 6: Fig. S6.** Comparison analysis and heatmap analysis of gut microbial taxonomic composition by metagenomics. (**a** and **b**) Comparison analysis of the relative abundances of gut microbial taxonomic compositions at phylum (**a**) and genus (**b**) levels by metagenomics, respectively. (**c** and **d**) Heatmap analysis of gut taxonomic compositions based on relative abundance at phylum (**c**) and genus (**d**) levels, respectively. The data was evaluated by Wilcox test analysis; n = 10 (CM-R) and n = 7 (DLY-R).

**Additional file 7: Fig. S7.** Analysis of gut bacterial diversity and taxonomic compositions by 16S rDNA gene amplicon survey. (**a**) Rarefaction curve analysis based on the Chao index. (**b**) Venn diagram analysis of bacterial ASVs. (**c**) Cluster tree analysis of gut bacterial beta diversity based on weighted unifrac distance. (**d**) Heatmap analysis of gut bacterial beta diversity based on the weighted Unifrac distance. (**e** and **f**) Alpha diversities evaluated using the Chao index (**e**) and Shannon index (**f**), respectively. (**g**) GraPhlAn analysis of gut bacterial compositions. (**h**) Cladogram of gut bacterial compositions using LEfSe.

**Additional file 8: Fig. S8.** Comparison analysis of gut bacterial taxonomic composition by 16S rDNA gene amplicon survey. (**a**) Comparison analysis of the relative abundances of gut bacterial taxonomic composition at phylum level by 16S rDNA gene amplicon survey. (**b**) Comparison analysis of the relative abundances of gut bacterial taxonomic composition at genus level by 16S rDNA gene amplicon survey. The data was evaluated by Wilcox test analysis; n = 10 (CM-R) and n = 7(DLY-R).

**Additional file 9: Fig. S9.** Analysis of KEGG pathways and COG functions in the bacterial communities predicted by PICRUSt2. (**a**) Analysis of differential KEGG pathways in gut bacterial communities between CM-R group and DLY-R group predicted by PICRUSt2. (**b**) Analysis of differential COG functions in gut bacterial communities between CM-R group and DLY-R group predicted by PICRUSt2. The data was evaluated by Wilcox test analysis; n = 10 (CM-R) and n = 7 (DLY-R).

**Additional file 10: Fig. S10.** Comparison analysis of gut bacterial communities in the FMT donor pigs and recipient GF mice. (**a**) PCoA of gut bacterial beta diversity based on weighted Unifrac distance (DLY-D, the donor Duroc × [Landrace × Yorkshire] pigs; CM-D, the donor Congjiang miniature pigs; DLY-R, the recipient GF mice that received the fecal microbiota from Duroc × [Landrace × Yorkshire] pigs; CM-R, the recipient GF mice that received the fecal microbiota from Congjiang miniature pigs). (**b**) Heatmap analysis of gut bacterial taxonomic compositions.

**Additional file 11: Fig. S11.** Analysis of intestinal immuohistochemical staining of E-Cadherin, ZO-1, and Connexin 43 proteins in SPF mice treated with *B. fragilis*. (**a**-**c**) Representative images of immuohistochemical staining of the jejunal E-Cadherin (**a**), ZO-1 (**b**), and Connexin 43 proteins (**c**). The experiments for negative control were performed by omitting the primary antibody.

**Additional file 12: Fig. S12.** Analysis of intestinal immuohistochemical staining of RegIIIγ protein in SPF mice treated with *B. fragilis*. (**a** and **b**) Representative images of immuohistochemical staining of the jejunal RegIIIγ protein (**a**) and mean optical density analysis of RegIIIγ (**b**). The experiments for negative control were performed by omitting the primary antibody. The data are presented as mean ± SEM (n = 5) and evaluated using Student's *t*-test; ns, not significant.

**Additional file 13: Fig. S13.** Analysis of intestinal histological morphology in SPF mice treated with *B. fragilis*. (**a**) Representative images of intestinal histological morphology by hematoxylin and eosin staining of duodenum, jejunum, and ileum, respectively (Ctrl, control; BF, *B. fragilis*). (**b**-**d**) Statistical analysis of the villus height (**b**), crypt depth (**c**), and the ratio of the villus height to the crypt depth (**d**). The data are presented as mean ± SEM (n = 5) and evaluated using two-way ANOVA; ns, not significant.

**Additional file 14: Fig. S14.** Effects of StemRegenin 1 on the 3-phenylpropionic acid-activated AhR signaling. (**a**) Representative western blotting of AhR, Lamin B1, β-actin, and CYP1A1 in the jejunal epithelium of mice (Ctrl, control; 3-PPA, 3-phenylpropionic acid; SR1, StemRegenin 1). (**b**-**d**) Quantitation of AhR levels (**b**) normalized to Lamin B1 levels. Quantitation of AhR (**c**) and CYP1A1 (**d**) levels normalized to β-actin levels. (**e**) Levels of IL-22 in the jejunal epithelium of mice. The data are presented as the mean ± SEM and evaluated by one-way ANOVA; n = 3 (**b**-**d**) and n = 8 (**e**); **p < 0.01; *p < 0.05; ns, not significant.

## Data Availability

The raw sequencing data has been deposited into China National GeneBank Sequence Archive (CNSA) of China National GeneBank DataBase (CNGBdb) with the accession numbers CNP0002106 and CNP0003730.
