# Correction: Gut microbiota-derived 3-phenylpropionic acid promotes intestinal epithelial barrier function via AhR signaling

**DOI:** 10.1186/s40168-023-01576-0

**Published:** 2023-05-20

**Authors:** Jun Hu, Jianwei Chen, Xiaojian Xu, Qiliang Hou, Jing Ren, Xianghua Yan

**Affiliations:** 1 State Key Laboratory of Agricultural Microbiology, Hubei Hongshan Laboratory, Frontiers Science Center for Animal Breeding and Sustainable Production, College of Animal Sciences and Technology, Huazhong Agricultural University, Wuhan, 430070 Hubei China; 2 The Cooperative Innovation Center for Sustainable Pig Production, Wuhan, 430070 Hubei China; 3 Hubei Provincial Engineering Laboratory for Pig Precision Feeding and Feed Safety Technology, Wuhan, 430070 Hubei China; 4 BGI Research-Qingdao, BGI, Qingdao, 266555 China

**Correction: Microbiome 11, 102 (2023)**

**https://doi.org/10.1186/s40168-023-01551-9**

Following publication of the original article [1], the author reported that the caption for additional file figures were not included in the article.

The original article has been updated to correct this.
