# Supplementary figures and images for: Gut microbiota-derived 3-phenylpropionic acid promotes intestinal epithelial barrier function via AhR signaling
Source: Microbiome. 2023 May 8;11:102. doi: 10.1186/s40168-023-01551-9 (PMC10165798; doi:10.1186/s40168-023-01551-9)

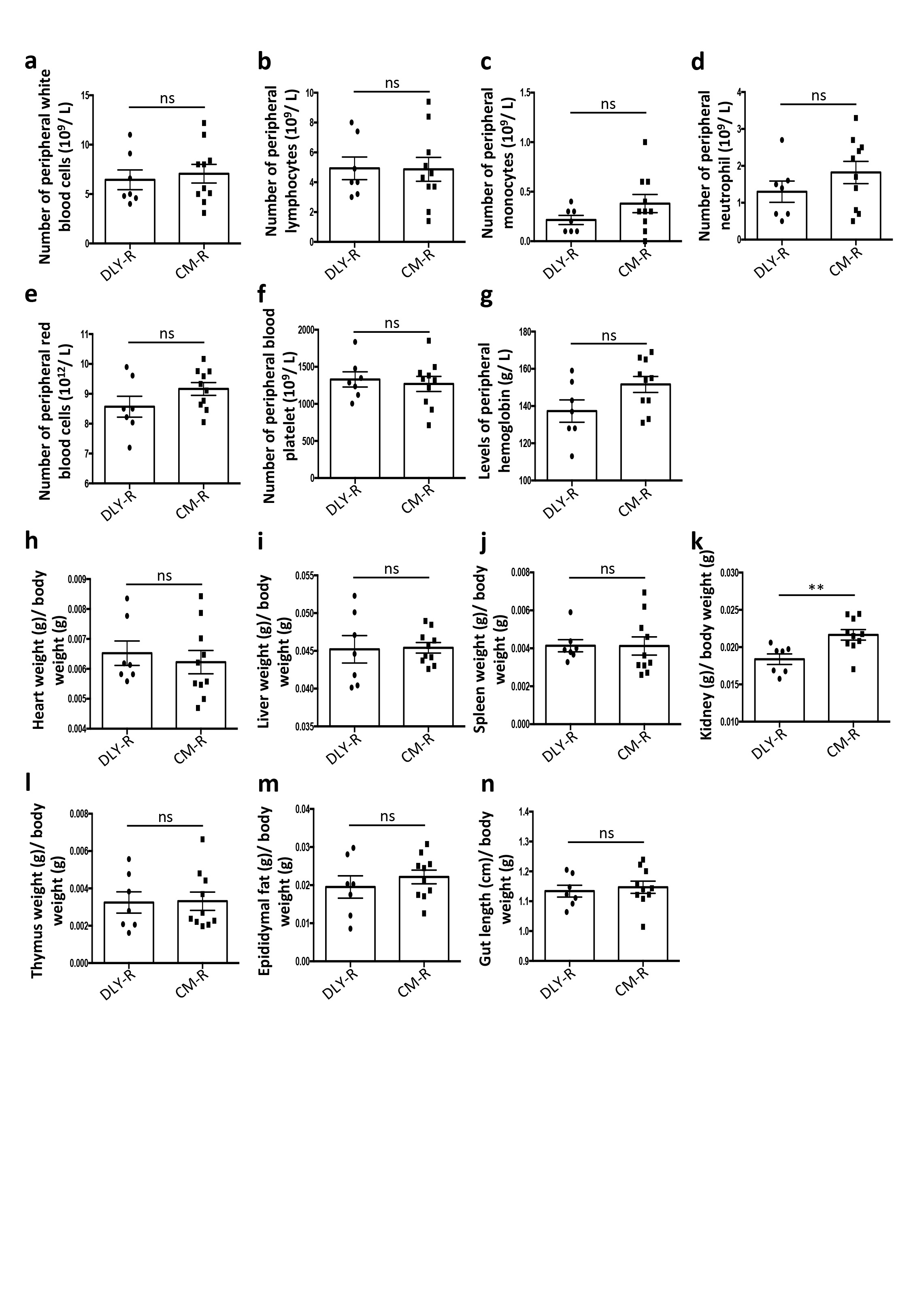

Supplement: Supplementary file 2 — Additional file 1: Fig. S1. Analyses of blood routine indices and organ indices in GF mice treated with FMT. (a-g) The number of peripheral white blood cells (a), lymphocytes (b), monocytes (c), neutrophils (d), red blood cells (e), and blood platelets (f) in mice and the levels of peripheral hemoglobin (g) in mice (CM-R, the recipient GF mice that received the fecal microbiota from Congjiang miniature pigs; DLY-R, the recipient GF mice that received the fecal microbiota from Duroc × [Landrace × Yorkshire] pigs). (h-n) The heart (h), liver (i), spleen (j), kidney (k), thymus (l), epididymal fat (m), and gut length indices (n) in mice. The data are presented as the mean ± SEM and evaluated using Student's t-test; n = 10 (CM-R) and n = 7 (DLY-R). **p < 0.01; ns, not significant. [file 40168_2023_1551_MOESM1_ESM.jpg]

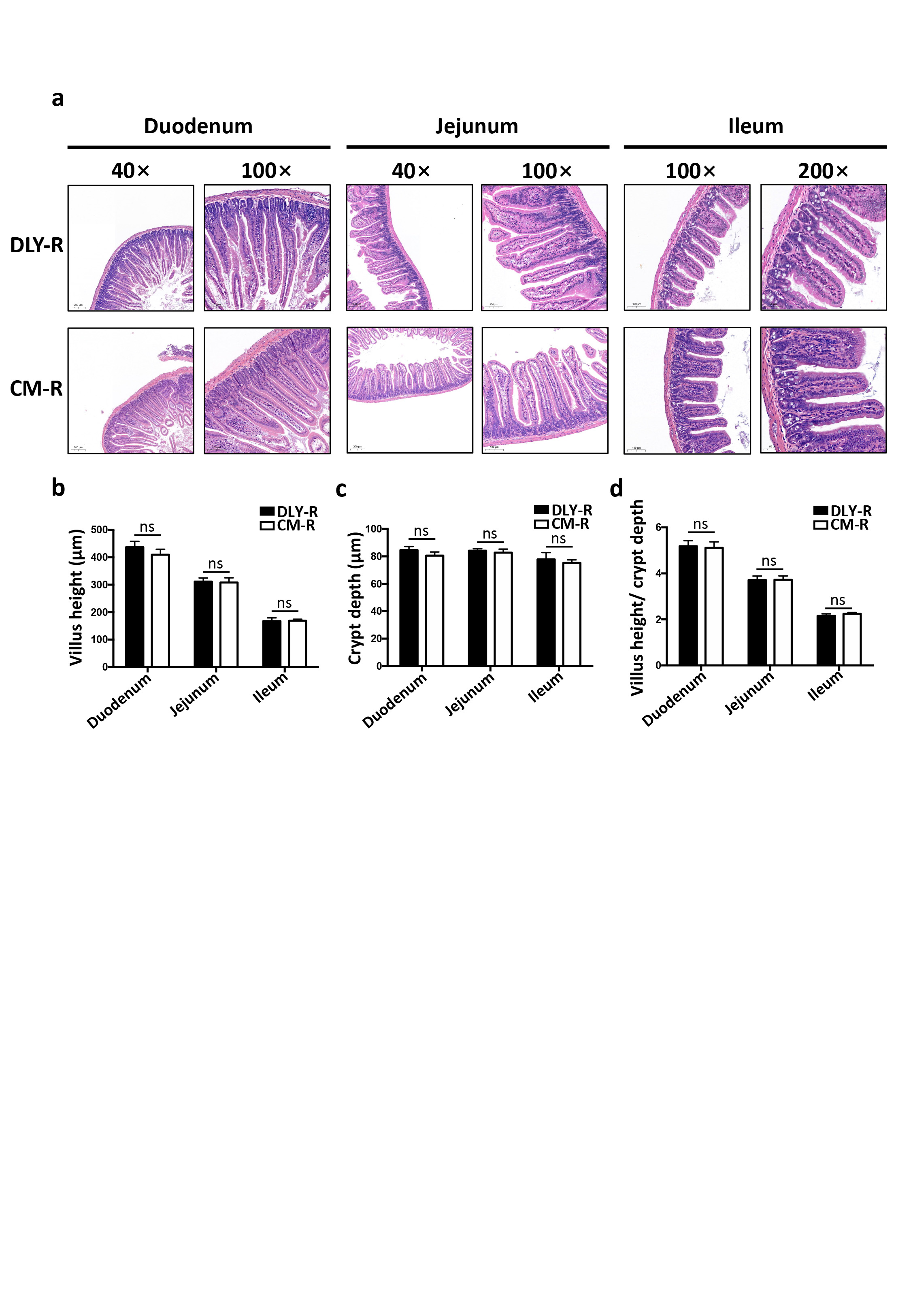

Supplement: Supplementary file 3 — Additional file 2: Fig. S2. Analysis of intestinal histological morphology in GF mice treated with FMT. (a) Representative images of intestinal histological morphology by hematoxylin and eosin staining of duodenum, jejunum, and ileum, respectively. (b-d) Statistical analysis of the villus height (b), crypt depth (c), and the ratio of the villus height to the crypt depth (d). The data are presented as mean ± SEM and evaluated by two-way analysis of variance (ANOVA); n = 10 (CM-R) and n = 7 (DLY-R); ns, not significant. [file 40168_2023_1551_MOESM2_ESM.jpg]

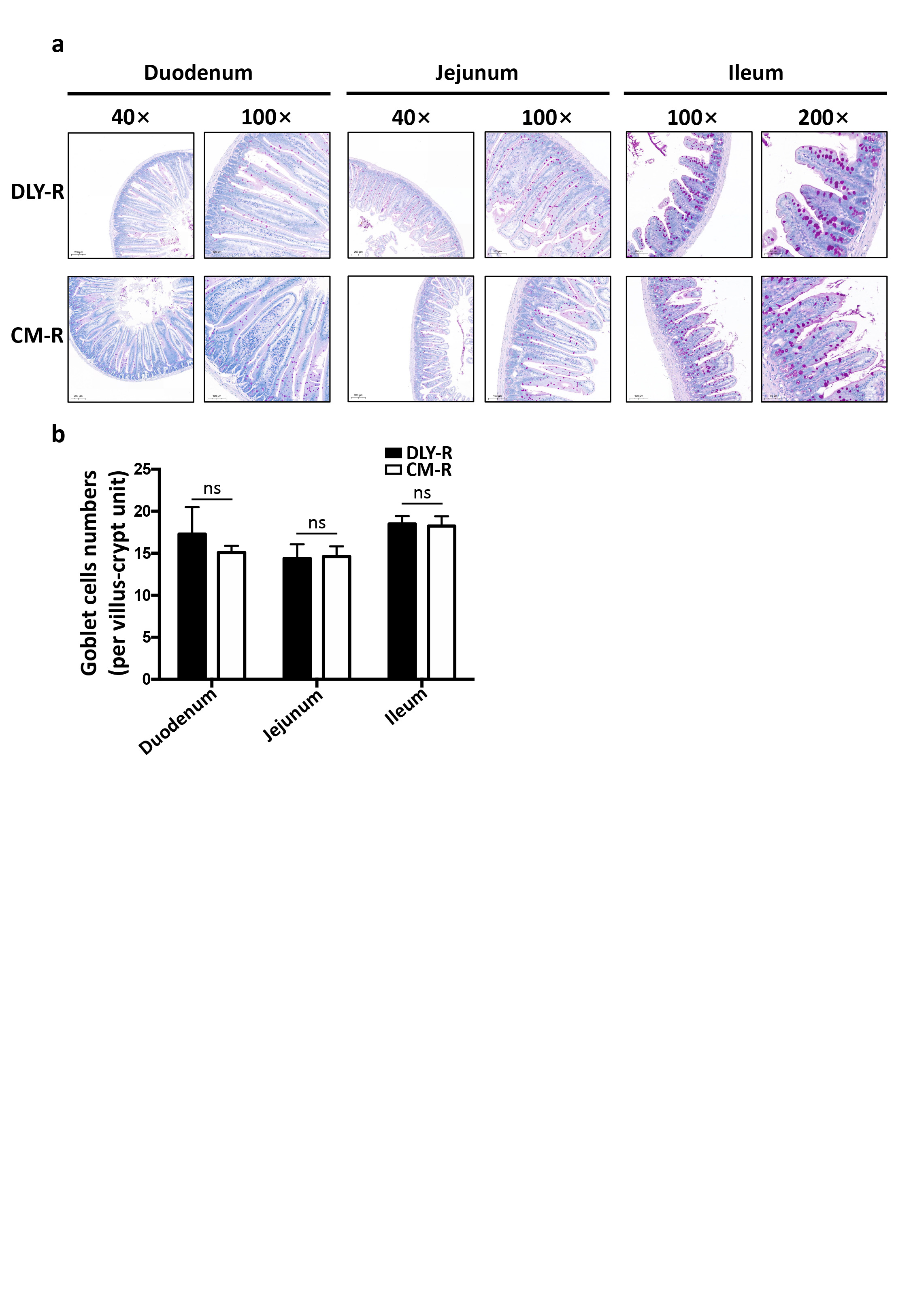

Supplement: Supplementary file 4 — Additional file 3: Fig. S3. Analysis of the numbers of intestinal goblet cells in GF mice treated with FMT. (a) Representative images of intestinal goblet cells stained with PAS staining of the duodenum, jejunum, and ileum, respectively. (b) Statistical analysis of goblet cell numbers in the duodenum, jejunum, and ileum, respectively. The data are presented as the mean ± SEM and evaluated by two-way ANOVA; n = 5; ns, not significant. [file 40168_2023_1551_MOESM3_ESM.jpg]

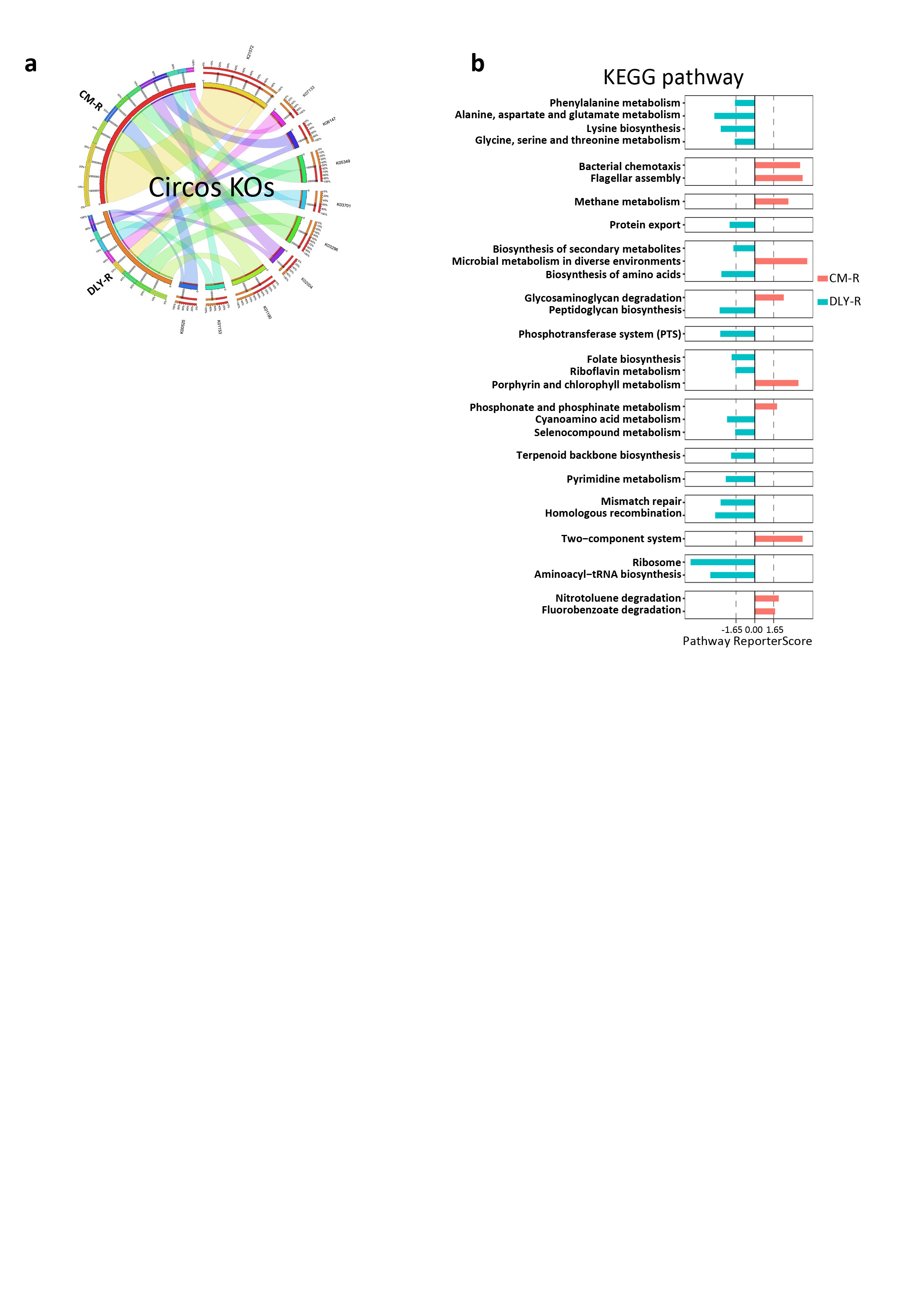

Supplement: Supplementary file 5 — Additional file 4: Fig. S4. Analysis of gut microbial functional profiles in GF mice treated with FMT by metagenomics. (a) Circos analysis of gut microbial KEGG orthologous groups (KOs). (b) Enrichment analysis of KEGG pathways in the gut microbiome. [file 40168_2023_1551_MOESM4_ESM.jpg]

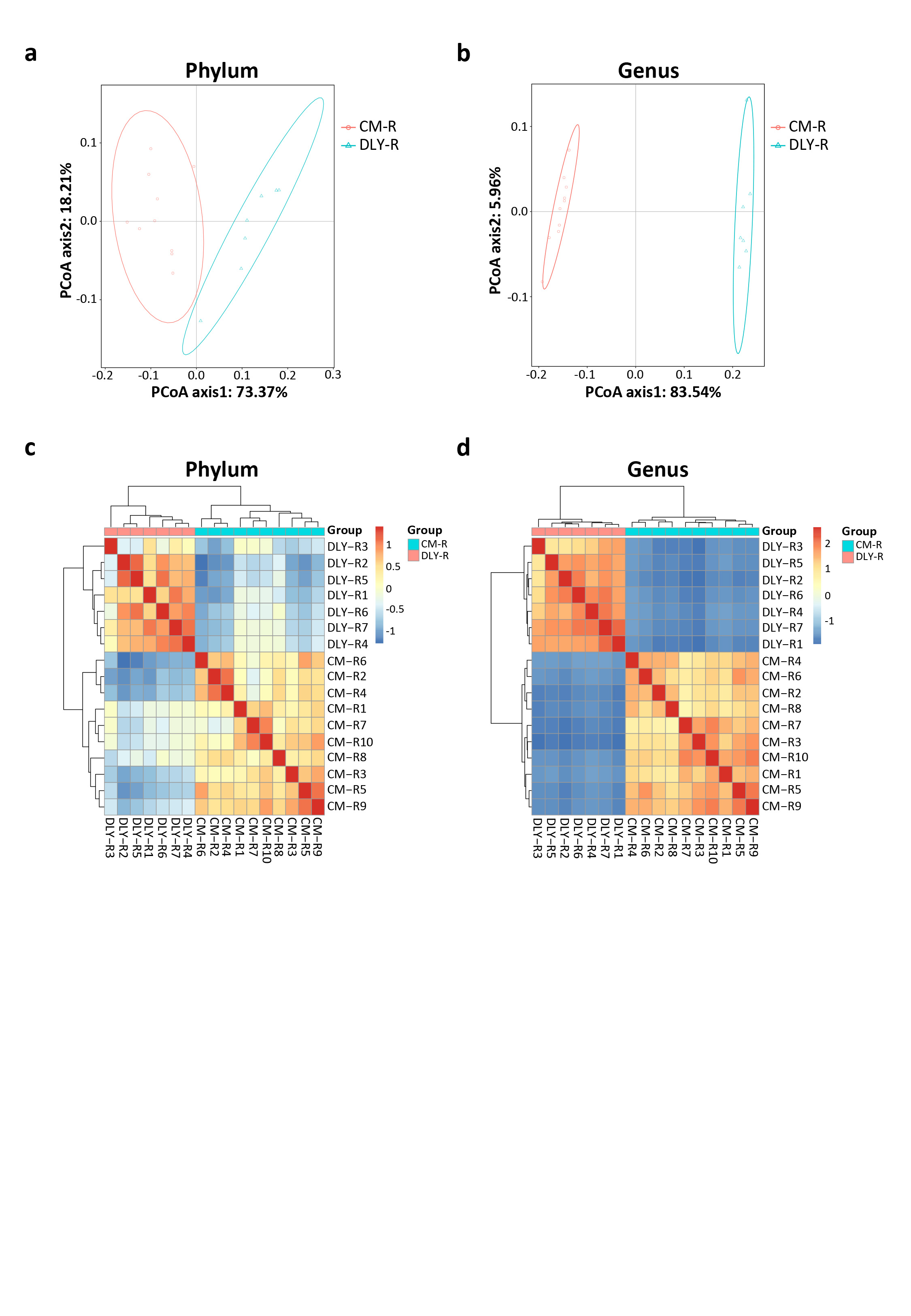

Supplement: Supplementary file 6 — Additional file 5: Fig. S5. Analysis of gut microbial taxonomic composition based on beta diversity by metagenomics. (a and b) PCoA of gut microbial taxonomic composition based on beta diversity at phylum (a) and genus (b) levels, respectively. (c and d) Heatmap analysis of gut taxonomic composition based on beta diversity at phylum (c) and genus (d) levels, respectively. [file 40168_2023_1551_MOESM5_ESM.jpg]

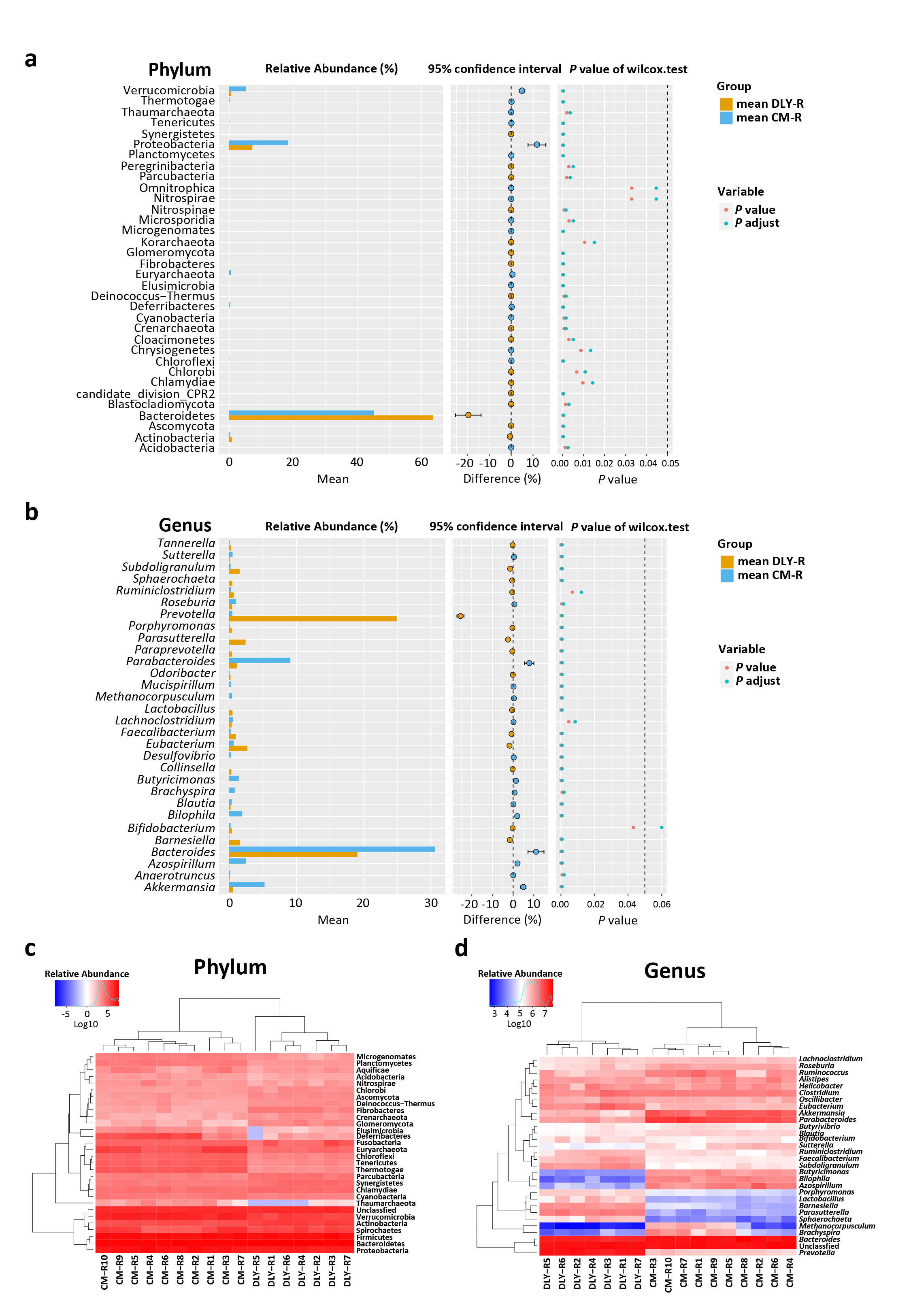

Supplement: Supplementary file 7 — Additional file 6: Fig. S6. Comparison analysis and heatmap analysis of gut microbial taxonomic composition by metagenomics. (a and b) Comparison analysis of the relative abundances of gut microbial taxonomic compositions at phylum (a) and genus (b) levels by metagenomics, respectively. (c and d) Heatmap analysis of gut taxonomic compositions based on relative abundance at phylum (c) and genus (d) levels, respectively. The data was evaluated by Wilcox test analysis; n = 10 (CM-R) and n = 7 (DLY-R). [file 40168_2023_1551_MOESM6_ESM.jpg]

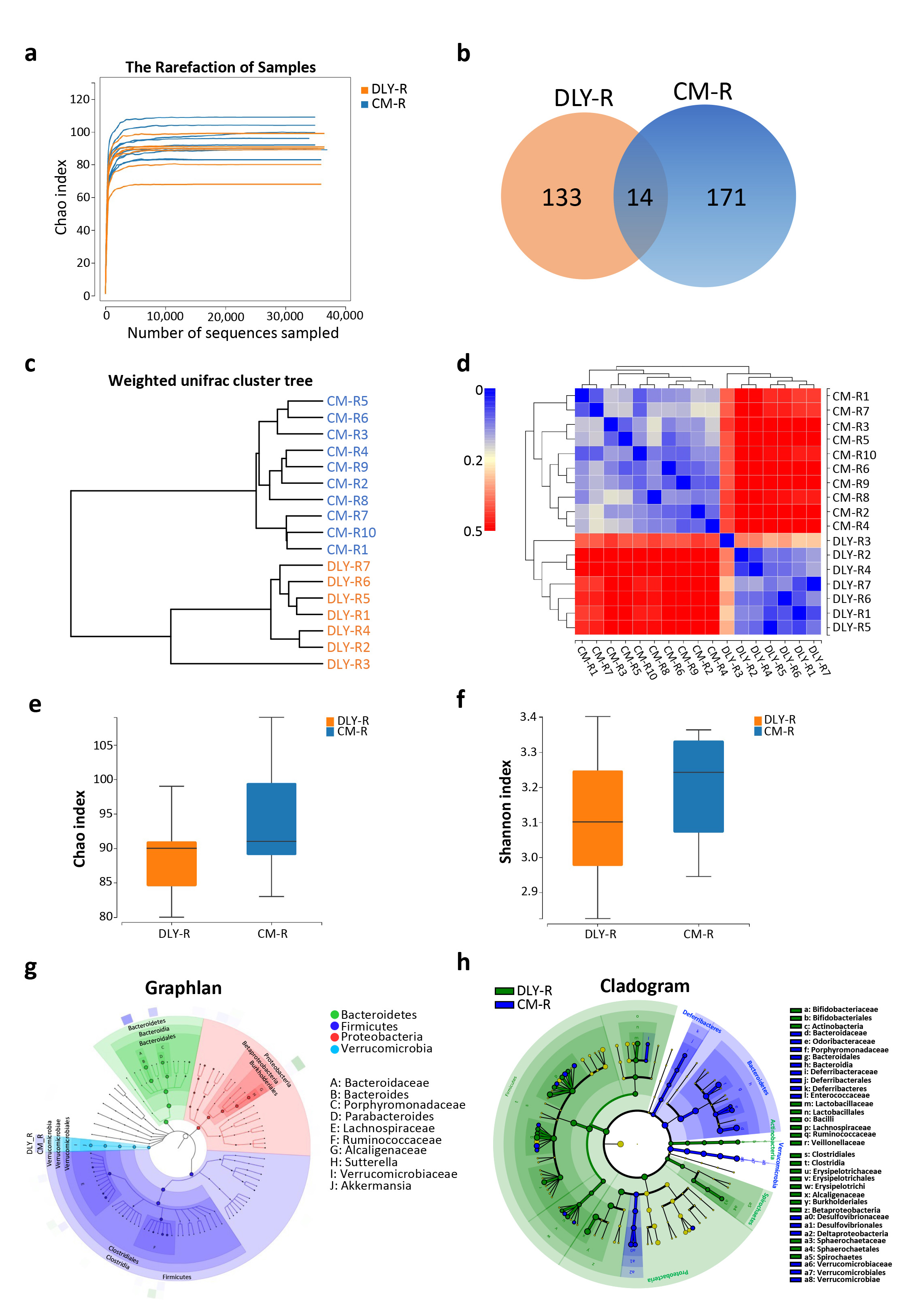

Supplement: Supplementary file 8 — Additional file 7: Fig. S7. Analysis of gut bacterial diversity and taxonomic compositions by 16S rDNA gene amplicon survey. (a) Rarefaction curve analysis based on the Chao index. (b) Venn diagram analysis of bacterial ASVs. (c) Cluster tree analysis of gut bacterial beta diversity based on weighted unifrac distance. (d) Heatmap analysis of gut bacterial beta diversity based on the weighted Unifrac distance. (e and f) Alpha diversities evaluated using the Chao index (e) and Shannon index (f), respectively. (g) GraPhlAn analysis of gut bacterial compositions. (h) Cladogram of gut bacterial compositions using LEfSe. [file 40168_2023_1551_MOESM7_ESM.jpg]

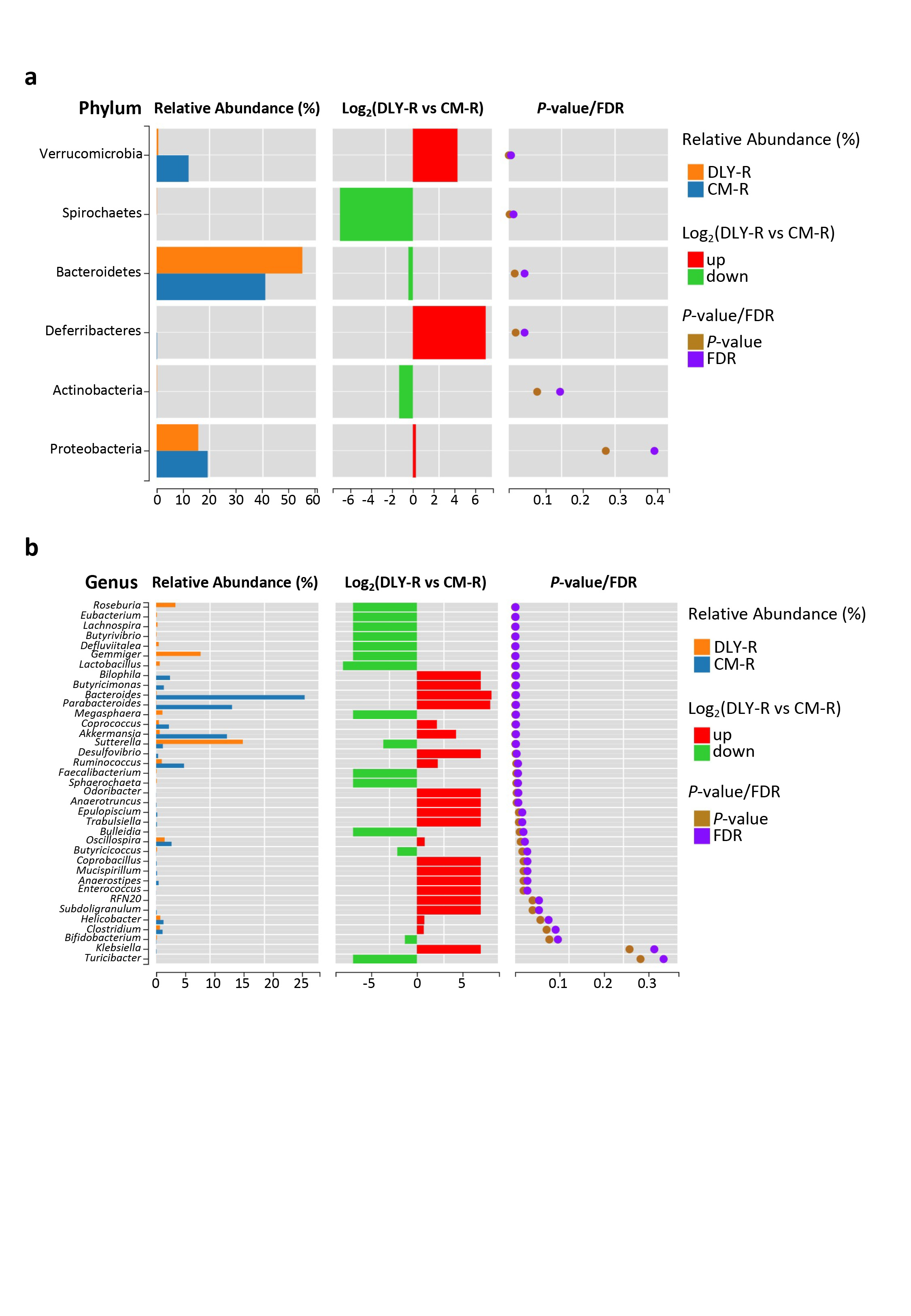

Supplement: Supplementary file 9 — Additional file 8: Fig. S8. Comparison analysis of gut bacterial taxonomic composition by 16S rDNA gene amplicon survey. (a) Comparison analysis of the relative abundances of gut bacterial taxonomic composition at phylum level by 16S rDNA gene amplicon survey. (b) Comparison analysis of the relative abundances of gut bacterial taxonomic composition at genus level by 16S rDNA gene amplicon survey. The data was evaluated by Wilcox test analysis; n = 10 (CM-R) and n = 7(DLY-R). [file 40168_2023_1551_MOESM8_ESM.jpg]

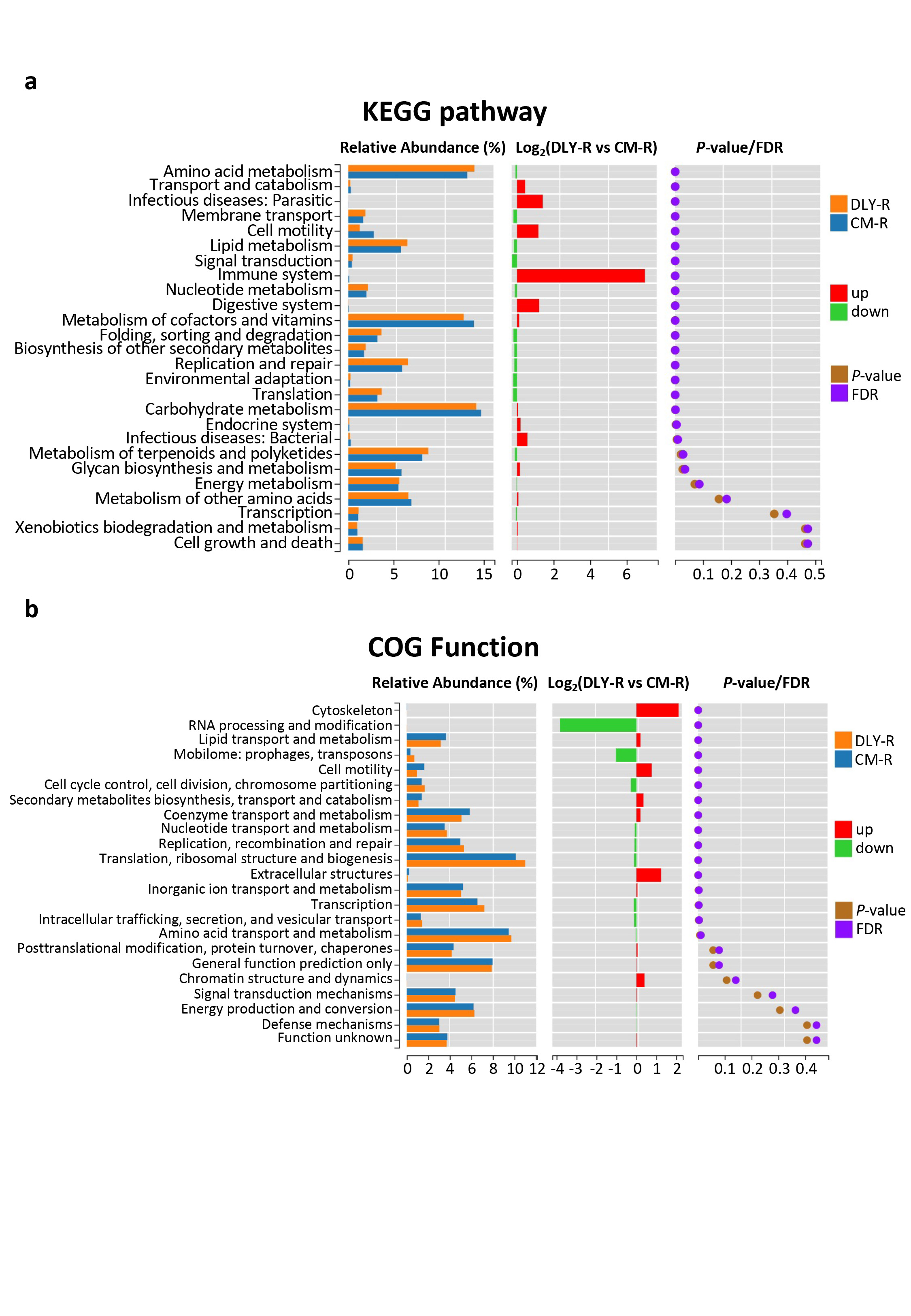

Supplement: Supplementary file 10 — Additional file 9: Fig. S9. Analysis of KEGG pathways and COG functions in the bacterial communities predicted by PICRUSt2. (a) Analysis of differential KEGG pathways in gut bacterial communities between CM-R group and DLY-R group predicted by PICRUSt2. (b) Analysis of differential COG functions in gut bacterial communities between CM-R group and DLY-R group predicted by PICRUSt2. The data was evaluated by Wilcox test analysis; n = 10 (CM-R) and n = 7 (DLY-R). [file 40168_2023_1551_MOESM9_ESM.jpg]

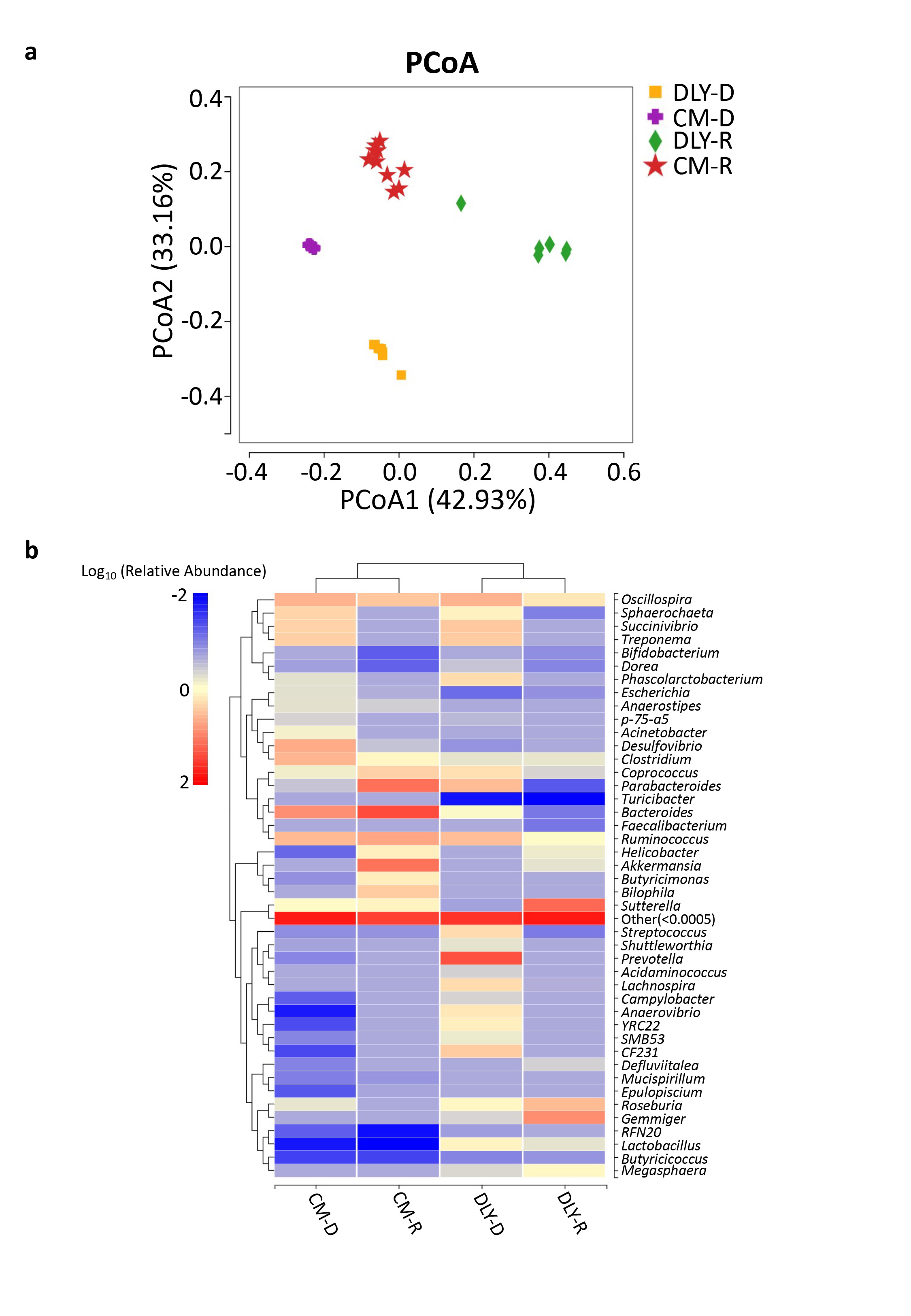

Supplement: Supplementary file 11 — Additional file 10: Fig. S10. Comparison analysis of gut bacterial communities in the FMT donor pigs and recipient GF mice. (a) PCoA of gut bacterial beta diversity based on weighted Unifrac distance (DLY-D, the donor Duroc × [Landrace × Yorkshire] pigs; CM-D, the donor Congjiang miniature pigs; DLY-R, the recipient GF mice that received the fecal microbiota from Duroc × [Landrace × Yorkshire] pigs; CM-R, the recipient GF mice that received the fecal microbiota from Congjiang miniature pigs). (b) Heatmap analysis of gut bacterial taxonomic compositions. [file 40168_2023_1551_MOESM10_ESM.jpg]

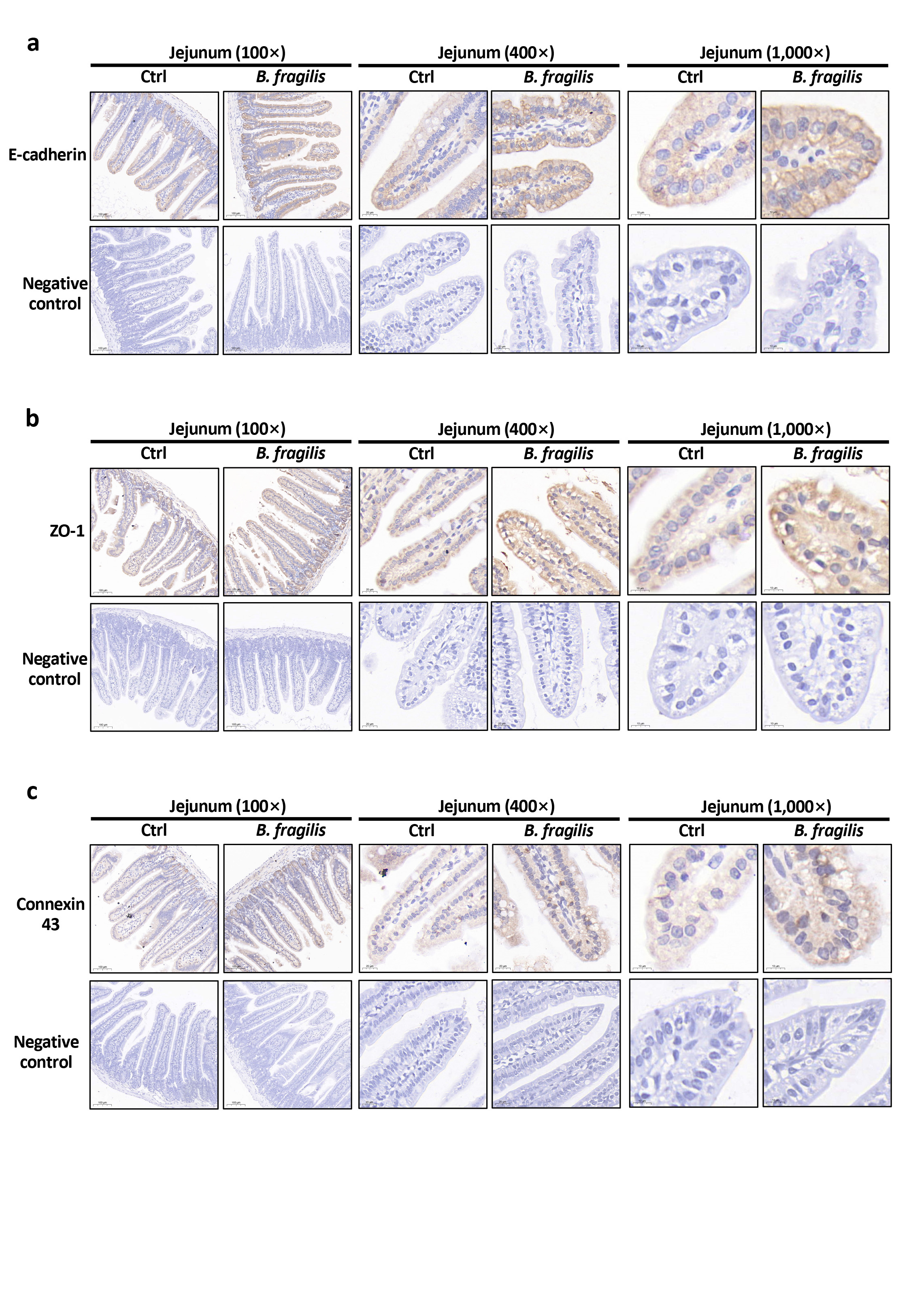

Supplement: Supplementary file 12 — Additional file 11: Fig. S11. Analysis of intestinal immuohistochemical staining of E-Cadherin, ZO-1, and Connexin 43 proteins in SPF mice treated with B. fragilis. (a-c) Representative images of immuohistochemical staining of the jejunal E-Cadherin (a), ZO-1 (b), and Connexin 43 proteins (c). The experiments for negative control were performed by omitting the primary antibody. [file 40168_2023_1551_MOESM11_ESM.jpg]

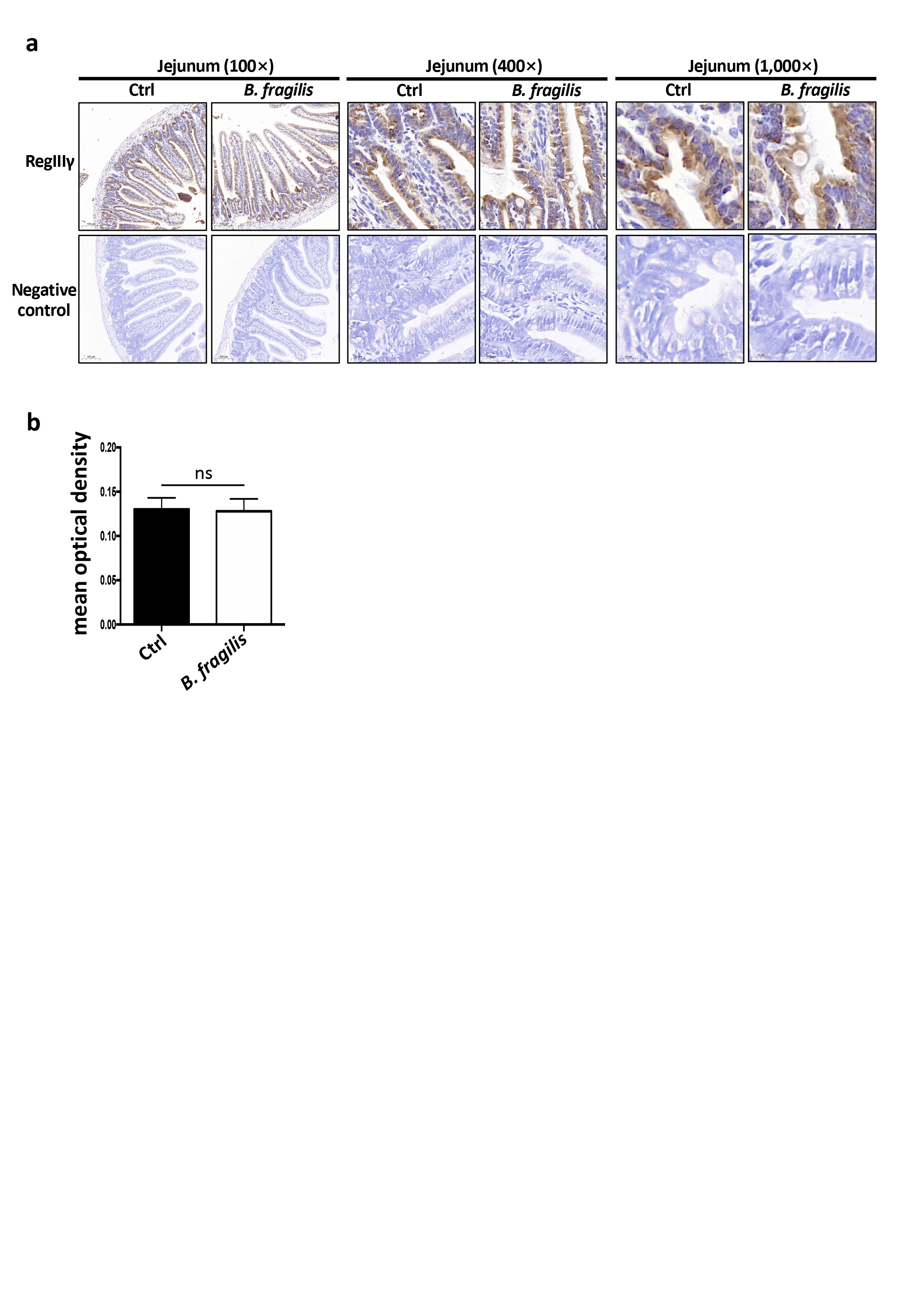

Supplement: Supplementary file 13 — Additional file 12: Fig. S12. Analysis of intestinal immuohistochemical staining of RegIIIγ protein in SPF mice treated with B. fragilis. (a and b) Representative images of immuohistochemical staining of the jejunal RegIIIγ protein (a) and mean optical density analysis of RegIIIγ (b). The experiments for negative control were performed by omitting the primary antibody. The data are presented as mean ± SEM (n = 5) and evaluated using Student's t-test; ns, not significant. [file 40168_2023_1551_MOESM12_ESM.jpg]

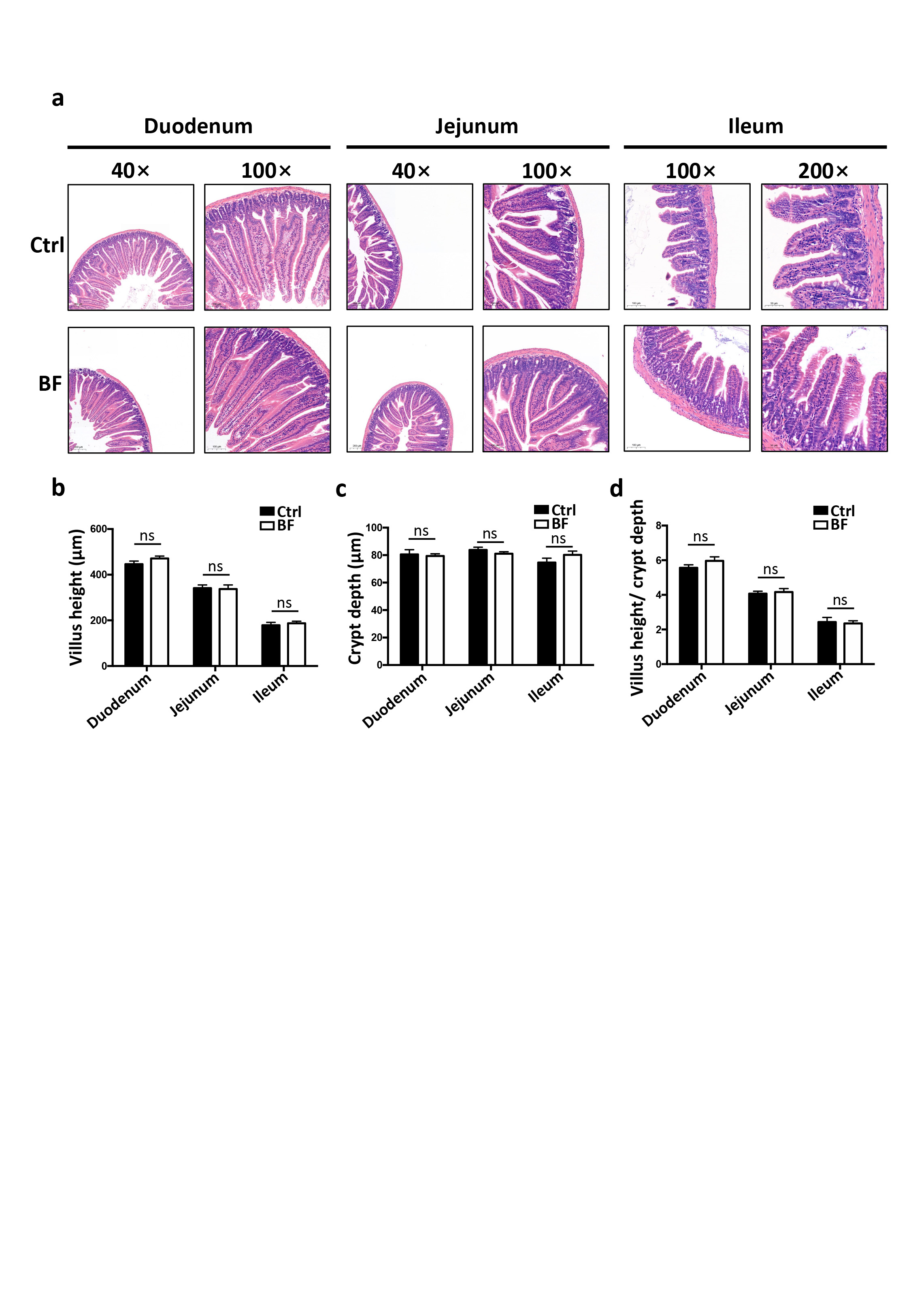

Supplement: Supplementary file 14 — Additional file 13: Fig. S13. Analysis of intestinal histological morphology in SPF mice treated with B. fragilis. (a) Representative images of intestinal histological morphology by hematoxylin and eosin staining of duodenum, jejunum, and ileum, respectively (Ctrl, control; BF, B. fragilis). (b-d) Statistical analysis of the villus height (b), crypt depth (c), and the ratio of the villus height to the crypt depth (d). The data are presented as mean ± SEM (n = 5) and evaluated using two-way ANOVA; ns, not significant. [file 40168_2023_1551_MOESM13_ESM.jpg]

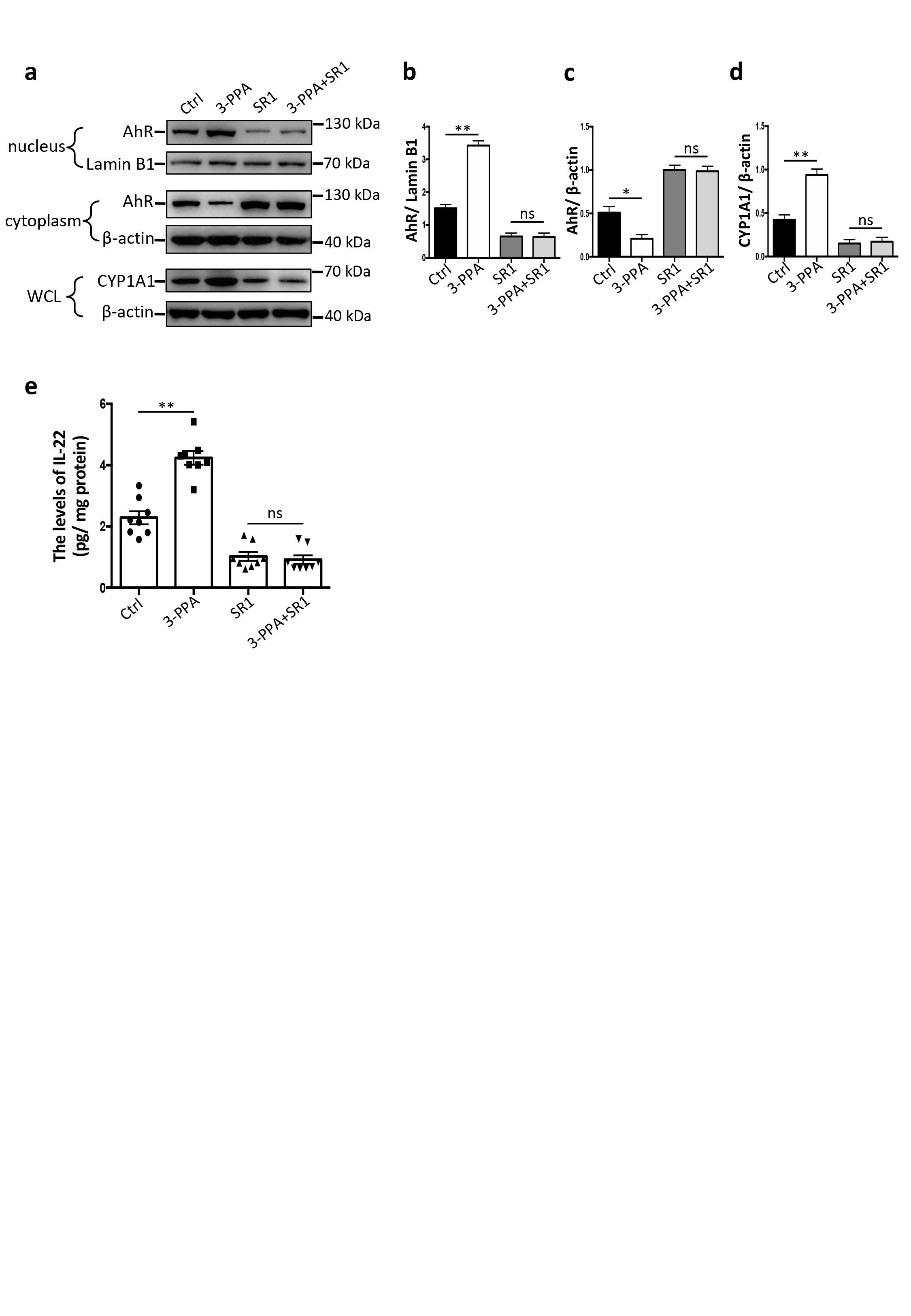

Supplement: Supplementary file 15 — Additional file 14: Fig. S14. Effects of StemRegenin 1 on the 3-phenylpropionic acid-activated AhR signaling. (a) Representative western blotting of AhR, Lamin B1, β-actin, and CYP1A1 in the jejunal epithelium of mice (Ctrl, control; 3-PPA, 3-phenylpropionic acid; SR1, StemRegenin 1). (b-d) Quantitation of AhR levels (b) normalized to Lamin B1 levels. Quantitation of AhR (c) and CYP1A1 (d) levels normalized to β-actin levels. (e) Levels of IL-22 in the jejunal epithelium of mice. The data are presented as the mean ± SEM and evaluated by one-way ANOVA; n = 3 (b-d) and n = 8 (e); **p < 0.01; *p < 0.05; ns, not significant. [file 40168_2023_1551_MOESM14_ESM.jpg]
